# AoI-Aware Optimization of Service Caching-Assisted Offloading and Resource Allocation in Edge Cellular Networks

**DOI:** 10.3390/s23063306

**Published:** 2023-03-21

**Authors:** Jialiang Feng, Jie Gong

**Affiliations:** The Guangdong Key Laboratory of Information Security Technology, School of Computer Science and Engineering, Sun Yat-Sen University, Guangzhou 510006, China

**Keywords:** edge computing, computation offloading, service caching, age of information, resource allocation

## Abstract

The rapid development of the Internet of Things (IoT) has led to computational offloading at the edge; this is a promising paradigm for achieving intelligence everywhere. As offloading can lead to more traffic in cellular networks, cache technology is used to alleviate the channel burden. For example, a deep neural network (DNN)-based inference task requires a computation service that involves running libraries and parameters. Thus, caching the service package is necessary for repeatedly running DNN-based inference tasks. On the other hand, as the DNN parameters are usually trained in distribution, IoT devices need to fetch up-to-date parameters for inference task execution. In this work, we consider the joint optimization of computation offloading, service caching, and the AoI metric. We formulate a problem to minimize the weighted sum of the average completion delay, energy consumption, and allocated bandwidth. Then, we propose the AoI-aware service caching-assisted offloading framework (ASCO) to solve it, which consists of the method of Lagrange multipliers with the KKT condition-based offloading module (LMKO), the Lyapunov optimization-based learning and update control module (LLUC), and the Kuhn–Munkres (KM) algorithm-based channel-division fetching module (KCDF). The simulation results demonstrate that our ASCO framework achieves superior performance in regard to time overhead, energy consumption, and allocated bandwidth. It is verified that our ASCO framework not only benefits the individual task but also the global bandwidth allocation.

## 1. Introduction

In recent decades, the Internet of things (IoT) has experienced rapid development and become ubiquitous in our daily lives. IoT devices have proliferated and evolved with advanced hardware architectures, and are being leveraged to create seamless networks that cover every corner of our globe [1]. Along with the development of IoT devices, a promising computing paradigm known as edge computing has arisen; this involves moving the location of computation from the central network to the network edge [2]. Moving the task execution from the cloud server to the multi-access edge computing (MEC) server (e.g., base station, access point) significantly alleviates the congestion of the core network and releases the burden of the cloud. Tasks with real-time requirements, computation-intensive characteristics, and high energy consumption (e.g., deep neural network (DNN)-based automatic license plate recognition) appear. Mobile devices where tasks are generated are constrained in terms of energy and computational capabilities (e.g., smartphones and unmanned aerial vehicles). Therefore, it is necessary to offload tasks to nearby MEC servers for remote execution [3], which is also known as computation offloading [4].

However, the exponential growth in the volume of offloaded data has led to increased traffic burdens on cellular networks, causing channel congestion. Under unstable network conditions, such as extremely high transmission latency, the performance of computation offloading can drastically decline. A caching policy [5] is proposed to tackle this issue by proactively storing the service in IoT devices, including MEC servers and mobile devices, to reduce the traffic of the cellular network. If an IoT device caches the service libraries and parameters, the task can be directly processed. Hence, the task processing time can dramatically reduce [6]. A DNN-based task is executed by a corresponding service package, consisting of reliable libraries and network parameters. Since the MEC server and mobile devices process distinct types of tasks, it is impractical to proactively cache all types of services due to storage limits. They only carry out caching whenever a task is required to be executed, and the caches are stored within a restricted time horizon.

Machine learning plays a significant role in the wireless network [7]. Considering a distributed machine learning scenario [8], the DNN is trained in a distributed manner. Then, the trained parameters of the DNN are assembled on an application server. The application server gathers all of the trained parameters and further trains a global DNN. Since the new data are generated from mobile devices, the trained parameters are updated ceaselessly and the global DNN is retrained based on the newly gathered parameters at the end of every global training round. Thus, the global DNN always reflects the up-to-date trained parameters. However, mobile devices may not fetch the latest parameters in every round. Hence, the cached DNN model may be outdated, which should be updated to keep the model fresh. To measure the freshness of the global service parameters at the MEC servers and the mobile devices, we introduce the concept of AoI [9], which is defined as the elapsed time since the generation of the latest received global service parameters response. The global service parameters are generated by training at the end of every global training round. When the MEC server or mobile device is required to execute inference tasks, it first checks whether fresh service parameters exist. If the service parameters are stale, the MEC server or mobile device needs to request the application server to fetch the up-to-date trained parameters for inference task execution.

### 1.1. Challenges

To realize distributed machine learning and service caching, the following challenges should be addressed:

#### 1.1.1. Cost of the Task

On the one hand, the inference task completion time needs to be less than its corresponding maximal tolerance deadline. Thus, minimizing the inference task completion time is necessary for real-time requirements. On the other hand, the inference tasks are generated on energy-constrained mobile devices, which carefully make the offloading decisions to minimize energy consumption. Therefore, it is challenging to minimize the cost of the inference task consisting of time delay and energy consumed.

#### 1.1.2. Bandwidth Consumption of the Application Server

If IoT devices fetch the latest service parameters from the application server, it utilizes the limited wireless bandwidth of cellular networks. Therefore, there is a trade-off between the fetching time and the total available bandwidth. If the application preferentially guarantees the fetching time, the remained bandwidth is not enough to serve other applications, and vice versa. Thus, the challenge of time and bandwidth trade-off needs to be addressed.

#### 1.1.3. Matching between Wireless Channels and IoT Devices

In a condition of limited bandwidth, the matching between the wireless bandwidth and IoT devices is significant enough to minimize the fetching time since an IoT device may experience diverse channel fading and co-channel interference on different wireless channels. Hence, it is the third challenge to match between wireless channels and IoT devices to further minimize the service fetching time.

### 1.2. Related Work

#### 1.2.1. Offloading with Cache

Some works make offloading decisions by considering the cache technology. In [10], an algorithm was devised by taking into account the multi-cast opportunity with cache in a multi-user scenario. A computing offloading and content caching model was proposed to reduce the time delay in the internet of vehicles in [11]. In [12], an optimal computing offloading and caching policy was designed to minimize the latency in a hybrid mobile system. In [13], an approximation collaborative computation offloading scheme and a game-theoretic collaborative computation offloading scheme were devised to achieve better offloading performance and scale well with the increasing computation task numbers. The above works do not consider the age of the cache, which may degrade the QoS.

#### 1.2.2. Cache of Data

In terms of data caching, existing works focus on frequently reused data to improve performance. In [14], a deep supervised learning method was adopted to make real-time decisions in a dynamic vehicle network. An online caching placement and prediction-based data pre-fetch method were designed in [15] to address the uncertainty of future task parameters. In [16], a cache deployment strategy in a large-scale Wi-Fi system was adopted to maximize the caching benefit and achieve better caching performance. In [17], a joint power allocation–caching problem was formulated to maximize the downlink performance in the caching FiWi network. However, these works do not take into account the caching of the service, which is crucial in the DNN-based task.

#### 1.2.3. Cache of Service

With respect to service caching, a few works consider caching services to enhance system efficiency. In [18], an online caching algorithm was proposed to minimize the overall computation delay. An extremely compelling (but much less studied) problem was studied in MEC-enabled dense cellular networks in [19]. In [20], an online service caching algorithm was devised to achieve the optimal worst-case competitive ratio under homogeneous task arrivals. In [21], a cache placement algorithm was adopted to minimize the data traffic forwarded to the remote cloud. The above-mentioned works only studied the cache and did not combine it with offloading.

#### 1.2.4. Age of Information

In regard to the age of information, some works focused on minimizing the AoI of the optimized goal. In [9], the concept of AoI was first proposed, and general methods were derived to calculate the age metric, which can be applied to broad types of service systems. Dynamic cache content update scheduling algorithms were designed to minimize the average AoI of the dynamic content delivered to the users in [22]. In [23], a dueling deep R-network-based status updating algorithm was proposed by combining the dueling deep Q-network and R-learning to minimize the average cost. In [24], an algorithm aimed to obtain an optimal trade-off between age and latency was adopted for the freshness-aware buffer update in a mobile edge scenario. However, these works did not leverage the AoI metric to improve the offloading performance in an edge system.

### 1.3. Contribution

In this paper, we consider an AoI-aware service caching-assisted offloading scenario. Our objective is to minimize the weighted sum of the average completion delay, energy consumption, and allocated bandwidth. We decompose the original problem into three subproblems: minimizing the average time overhead cost and energy consumption of inference tasks, minimizing the required average bandwidth, and minimizing the fetching time of responding IoT devices. Furthermore, to solve the subproblems, we propose the AoI-aware service caching-assisted offloading framework (ASCO) to deal with them, which consists of three modules: the method of Lagrange multipliers with the KKT condition-based offloading module (LMKO), the Lyapunov optimization-based learning and update control module (LLUC), and the Kuhn–Munkres (KM) algorithm-based channel-division fetching module (KCDF). Simulation results show that our ASCO framework achieves superior performance compared to other baseline combinations in terms of time overhead, energy consumption, and allocated bandwidth. The main contributions of the paper are summarized as

1. To minimize the average time overhead cost and energy consumption of inference tasks, we transform the problem into a Lagrangian dual problem. Then, we propose the LMKO module based on the method of Lagrange multipliers with Karush–Kuhn–Tucker (KKT) conditions to make an optimal offloading decision.
2. To minimize the required average bandwidth, we transform the problem into a Lyapunov plus penalty problem by minimizing the total required bandwidth while keeping the requesting data queue backlog stable. Further, we propose the LLUC module based on the Lyapunov optimization to derive an optimal dequeued rate.
3. To minimize the fetching time of IoT devices, we consider the problem of finding the perfect matching by maximizing the sum of the link weights in the equalling subgraph. Moreover, we propose the KCDF module based on the KM algorithm to obtain the optimal matching decision.

The novelty of the paper consists of three aspects. First we propose an AoI-aware service caching-assisted offloading scenario, which has not been considered in the literature. This scenario takes into account the service caching in distributed machine learning, including the service libraries and parameters. It is a popular technology and worthy to be investigated. We also consider the freshness of the service caching for the computation offloading. Existing works omit the AoI of the caching, especially the service caching, which degrades the offloading performance. We aim to minimize the costs from both the mobile device side and the global perspective. Then, we propose the novel ASCO framework, including three modules. The proposed algorithm outperforms the existing baselines.

The rest of the paper is organized as follows. We elaborate on the system module in Section 2. An analysis of the formulated problems is detailed in Section 3. Section 4 presents the proposed solution. The evaluation simulation is described in Section 5, followed by the conclusion in Section 6.

## 2. System Model

**Figure 1:**
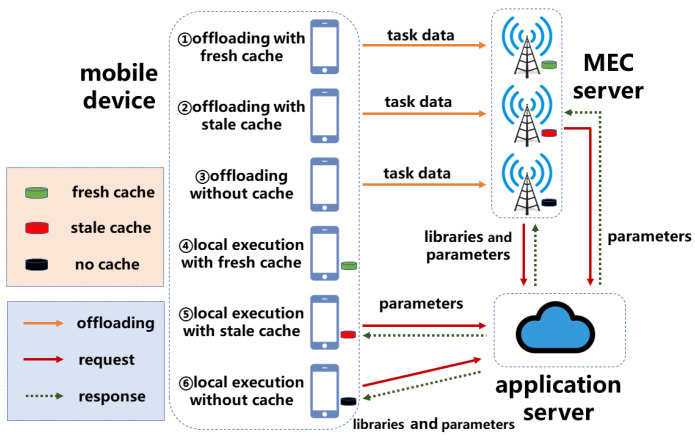
Schematics of the offloading cases.

We consider an AoI-aware service caching asymmetric network consisting of heterogeneous mobile devices and MEC servers in Figure 1. A set of |N| mobile devices indexed by *n* is denoted as N={1,⋯,|N|}, e.g., smartphones and intelligent vehicles. A set of |M| MEC servers indexed by *m* is denoted as M={1,⋯,|M|}, e.g., access points and base stations. Since an AI-based inference task generated from the mobile device is computationintensive and has real-time requirements, the mobile device with constrained computation capability needs to offload the inference task to the MEC server with sufficient computation resources. On the one hand, the inference task is processed by the corresponding service. For instance, an image recognition inference task is inferred by a DNN service running in service libraries, e.g., machine learning frameworks. On the other hand, caching data can alleviate the transmission traffic during the offloading and include content caching and service caching.

Considering a distributed machine learning scenario, an application server periodically trains an up-to-date DNN and then distributes it to the MEC servers and mobile devices, among which the inference task data are hardly reusable while the DNN service is frequently reusable. Thus, different from cashing the inference task data, caching the DNN service significantly reduces the transmission time. Note that the DNN service consists of the service libraries and service parameters. Since a DNN with the latest parameters owns better inference accuracy based on the periodical training, the service parameters should be updated when it has a new version. The service libraries are static and are only transmitted once for caching while the service parameters are dynamic. We define the AoI as the elapsed time since the generation of the latest received service parameters at MEC servers or mobile devices, which measures the freshness of service parameters. If the AoI of service parameters is less than the periodical training round, then the parameters are considered to be the latest version and fresh enough to be used for inference. Otherwise, since a new version is generated at the application server, the parameters are stale and need to be updated to the latest version. Note that mobile devices do not hold the AoI information on the side of MEC servers due to privacy concerns and transmission overhead.

### 2.1. Task Model

Considering a time-slotted system, a set of |T| timeslots indexed by *t* is denoted as T={1,⋯,|T|}. The inference task generated from the mobile device *n* at timeslot *t* is denoted as $k_{n}\left(t\right)$. A set of |J| service types indexed by *j* is denoted as J={1,⋯,|J|}. For instance, plate image recognition and face recognition are distinct types of services. Each inference task has a corresponding service type; the relationship is represented as follows: $x_{k_{n}\left(t\right),j}^{\mathrm{typ}}=1$ if $k_{n}\left(t\right)$ is of type *j*; otherwise, $x_{k_{n}\left(t\right),j}^{\mathrm{typ}}=0$. This satisfies the condition that each inference task can only be executed by a service of a certain type, at most: $\sum_{j\in J}x_{k_{n}\left(t\right),j}^{\mathrm{typ}}=1$.

The inference tasks are computation-intensive and have maximum time tolerance. We define the inference task profile of $k_{n}\left(t\right)$ as $\left(d_{k_{n}\left(t\right)},c_{k_{n}\left(t\right)},T_{k_{n}\left(t\right)}^{\max}\right)$, where $d_{k_{n}\left(t\right)}$ is the inference task input size, $c_{k_{n}\left(t\right)}$ is the task computation amount, and $T_{k_{n}\left(t\right)}^{\max}$ is the task maximum completion tolerance deadline. Take the task image recognition as an example, $d_{k_{n}\left(t\right)}$ is the image bit size, and $c_{k_{n}\left(t\right)}$ represents the required CPU cycles of the DNN service. $T_{k_{n}\left(t\right)}^{\max}$ is the image recognition deadline, meaning that the inference task processing delay cannot exceed the tolerance time.

### 2.2. Communication Model

The application server, mobile devices, and MEC servers mutually communicate under the cellular network. Based on the Shannon theory, the transmission rate between the mobile device *n* and MEC server *m* can be referred to as

(1) $$r_{n,m}\left(t\right)=b_{n,m}\left(t\right)\log_{2}\left(1+\frac{p_{n,m}\left(t\right)h_{n,m}\left(t\right)}{\sigma^{2}+I_{n,m}\left(t\right)}\right),$$

where $b_{n,m}\left(t\right)$ is the allocation bandwidth, $p_{n,m}\left(t\right)$ means the transmission power from *n* to *m*, $h_{n,m}\left(t\right)$ is the channel gain, $\sigma^{2}$ represents the additive white Gaussian noise, and $I_{n,m}\left(t\right)$ is the co-channel interference that mobile device *n* suffers on the cellular channel, respectively. The transmission power affects the achievable spectral efficiency, and a highly allocated bandwidth can lead to an efficient transmission rate. The channel gain between each one varies due to mobility. Since mobile devices are energy-constrained, the transmission power has an upper bound: $p_{n,m}\left(t\right)\leq p^{\max}$, where $p^{\max}$ is the maximum transmission power.

Moreover, the uploading time of inference task $k_{n}\left(t\right)$ from the mobile device *n* to the MEC server *m* can be calculated as

(2) $$T_{n,m,k_{n}\left(t\right)}^{\mathrm{upl}}=x_{m,k_{n}\left(t\right)}^{\mathrm{exe}}\left(t\right)\frac{d_{k_{n}\left(t\right)}}{r_{n,m}\left(t\right)},$$

where $x_{m,k_{n}\left(t\right)}^{\mathrm{exe}}\left(t\right)$ is the offloading decision and defined as $x_{m,k_{n}\left(t\right)}^{\mathrm{exe}}\left(t\right)=1$ if $k_{n}\left(t\right)$ is offloaded to *m*; otherwise, $x_{m,k_{n}\left(t\right)}^{\mathrm{exe}}\left(t\right)=0$. It satisfies: $\sum_{m\in M}x_{m,k_{n}\left(t\right)}^{\mathrm{exe}}\left(t\right)\leq 1$, meaning that each inference task is offloaded to one MEC server at most.

The transmission rate from the application server to the MEC server $r_{0,m}\left(t\right)$ and the transmission rate from the application server to the mobile device $r_{0,n}\left(t\right)$ can be similarly calculated with (1).

### 2.3. Caching Model

For the purpose of alleviating the transmission traffic, the MEC servers and mobile devices have to cache the DNN service in their caching storage if they have no corresponding cache. Let $d_{j}^{\mathrm{lib}}$ be the library size and $d_{j}^{\mathrm{par}}$ be the parameter size of service type *j*, respectively.

Therefore, the fetching time for the DNN service of type *j* to the MEC server *m* can be calculated as

(3) $$T_{0,m,j}^{\mathrm{cac}}=T_{0,m,j}^{\mathrm{lib}}+T_{0,m,j}^{\mathrm{par}}=\left(1-x_{m,j}^{\mathrm{cac}}\left(t\right)\right)\left(\frac{d_{j}^{\mathrm{lib}}}{r_{0,m}\left(t\right)}+\frac{d_{j}^{\mathrm{par}}}{r_{0,m}\left(t\right)}\right),$$

where $T_{0,m,j}^{\mathrm{lib}}$ and $T_{0,m,j}^{\mathrm{par}}$ are the fetching times of the service libraries and parameters at the MEC servers, respectively, and $x_{m,j}^{\mathrm{cac}}\left(t\right)$ is the service caching placement decision at the MEC server *m*, defined as $x_{m,j}^{\mathrm{cac}}\left(t\right)=1$ if *j* is cached in *m*; otherwise, $x_{m,j}^{\mathrm{cac}}\left(t\right)=0$. Similarly, the fetching time for the DNN service of type *j* to mobile device *n* can be represented as $T_{0,n,j}^{\mathrm{cac}}$.

Due to the limited caching capacity of the MEC server, there is a constraint on the storage cache:

(4) $$\sum_{j\in J}x_{m,j}^{\mathrm{cac}}\left(t\right)\left(d_{j}^{\mathrm{lib}}+d_{j}^{\mathrm{par}}\right)\leq d_{m}^{\max},$$

where the total DNN service size of all types cannot exceed the storage upper bound $d_{m}^{\max}$. The total DNN service size in mobile devices has a similar constraint.

Fresh parameters can effectively infer the DNN task with the satisfied performance. To measure the freshness of the DNN service parameters, we introduce the concept of AoI to quantify the age in the MEC server *m*:

(5) $$\Delta_{m,j}\left(t\right)=t-t_{j},$$

where $t_{j}$ is the timeslot of the latest periodical training of service type *j*. The same calculation of $\Delta_{n,j}\left(t\right)$ is in mobile devices. The updating mechanism at the MEC server *m* can be defined as $\Delta_{m,j}\left(t\right)=\frac{d_{j}^{\mathrm{par}}}{r_{0,m}\left(t\right)}$ if fetching ends at timeslot *t*; otherwise, $\Delta_{m,j}\left(t\right)=\Delta_{m,j}\left(t-1\right)+1$, and at mobile device *n*: $\Delta_{n,j}\left(t\right)=\frac{d_{j}^{\mathrm{par}}}{r_{0,n}\left(t\right)}$ if fetching ends at timeslot *t*; otherwise, $\Delta_{n,j}\left(t\right)=\Delta_{n,j}\left(t-1\right)+1$. Since the DNN is trained periodically in the application server, the DNN training round of type *j* can be denoted as $T_{j}^{\mathrm{int}}$. If the AoI of the parameters is less than the training round, the parameters can be regarded as fresh parameters. Let $x_{m,j}^{\mathrm{fre}}\left(t\right)$ be the service parameter freshness status, defined as follows: $x_{m,j}^{\mathrm{fre}}\left(t\right)=1$ if $\Delta_{m,j}\left(t\right)<T_{j}^{\mathrm{int}}$; otherwise, $x_{m,j}^{\mathrm{fre}}\left(t\right)=0$, and $x_{n,j}^{\mathrm{fre}}\left(t\right)=1$ if $\Delta_{n,j}\left(t\right)<T_{j}^{\mathrm{int}}$; otherwise, $x_{n,j}^{\mathrm{fre}}\left(t\right)=0$. If $x_{m,j}^{\mathrm{fre}}\left(t\right)=0$ or $x_{n,j}^{\mathrm{fre}}\left(t\right)=0$, the MEC server or the mobile device is required to fetch an up-to-date version of the service parameters from the application server; the fetching times are $T_{0,m,j}^{\mathrm{par}}$ and $T_{0,n,j}^{\mathrm{par}}$, respectively.

### 2.4. Execution Model

In terms of execution, the inference task is executed under the existence of the corresponding service. If there is no DNN service caching at the MEC server or mobile device, they are required to fetch a DNN service cache and then further carry out the execution. After fetching the service caching, the execution delay of inference task $k_{n}\left(t\right)$ at the MEC server *m* is calculated as

(6) $$T_{m,k_{n}\left(t\right)}^{\mathrm{exe}}=x_{m,k_{n}\left(t\right)}^{\mathrm{exe}}\left(t\right)\frac{c_{k_{n}\left(t\right)}}{f_{m}\left(t\right)},$$

where $f_{m}\left(t\right)$ is the computation capability of the MEC server *m*. Likewise, the execution delay at the mobile device *n* is calculated as

(7) $$T_{n,k_{n}\left(t\right)}^{\mathrm{exe}}=\left(1-\sum_{m\in M}x_{m,k_{n}\left(t\right)}^{\mathrm{exe}}\left(t\right)\right)\frac{c_{k_{n}\left(t\right)}}{f_{n}\left(t\right)},$$

where $f_{n}\left(t\right)$ is the constant computation capability of the mobile device *n*. Here, the computation capability of a mobile server is less than a MEC server, and $f_{m}\left(t\right)$ has an upper bound: $f_{n}\left(t\right)<f_{m}\left(t\right)\leq f^{\max}$, where $f^{\max}$ is the maximum of the computation capability.

### 2.5. Energy Model

From the perspective of energy consumption, we focus on the energy of mobile devices since they usually have batteries of limited capacity while the MEC server is connected to the power grid. Hence, the energy consumed for the local execution of the mobile device *n* can be calculated as

(8) $$E_{n,k_{n}\left(t\right)}^{\mathrm{exe}}=\left(1-\sum_{m\in M}x_{m,k_{n}\left(t\right)}^{\mathrm{exe}}\left(t\right)\right)\mu c_{k_{n}\left(t\right)}f_{n}^{2}\left(t\right),$$

where μ refers to the effective switched capacitance.

In the case of offloading, the energy consumption of the mobile device only includes the uploading energy, calculated as

(9) $$E_{n,m,k_{n}\left(t\right)}^{\mathrm{upl}}=x_{m,k_{n}\left(t\right)}^{\mathrm{exe}}\left(t\right)p_{n,m}\left(t\right)\frac{d_{k_{n}\left(t\right)}}{r_{n,m}\left(t\right)}.$$

Energy consumption is another crucial metric of mobile devices. The cost of the mobile device consists of the time delay and energy consumption with distinct emphasis.

### 2.6. Cost Model

At timeslot *t*, the mobile device *n* with the generated DNN inference task $k_{n}\left(t\right)$ can make an offloading decision to process the task. According to the service caching placement decision and service parameter freshness status, the cost of the mobile device can be divided into the following cases, as seen in Figure 1.

#### 2.6.1. Case 1: Offloading with Fresh Cache

First, in case 1, the mobile device offloads the inference task to the MEC server with caching service libraries and fresh parameters. The combination of the decision and status satisfies: $x_{k_{n}\left(t\right),1}\left(t\right)=x_{m,k_{n}\left(t\right)}^{\mathrm{exe}}\left(t\right)x_{k_{n}\left(t\right),j}^{\mathrm{typ}}x_{m,j}^{\mathrm{cac}}\left(t\right)x_{m,j}^{\mathrm{fre}}\left(t\right)=1$. The total time delay, in this case, can be calculated as $T_{k_{n}\left(t\right),1}=T_{n,m,k_{n}\left(t\right)}^{\mathrm{upl}}+T_{m,k_{n}\left(t\right)}^{\mathrm{exe}}$. In addition, the total energy consumption of the mobile device is represented as $E_{k_{n}\left(t\right),1}=E_{n,m,k_{n}\left(t\right)}^{\mathrm{upl}}$.

#### 2.6.2. Case 2: Offloading with Stale Cache

In Case 2, the mobile device offloads the inference task to the MEC server with caching service libraries and stale parameters. The combination of the decision and status satisfies: $x_{k_{n}\left(t\right),2}\left(t\right)=x_{m,k_{n}\left(t\right)}^{\mathrm{exe}}\left(t\right)x_{k_{n}\left(t\right),j}^{\mathrm{typ}}x_{m,j}^{\mathrm{cac}}\left(t\right)\left(1-x_{m,j}^{\mathrm{fre}}\left(t\right)\right)=1$. The total time delay, in this case, can be calculated as $T_{k_{n}\left(t\right),2}=T_{n,m,k_{n}\left(t\right)}^{\mathrm{upl}}+T_{0,m,j}^{\mathrm{par}}+T_{m,k_{n}\left(t\right)}^{\mathrm{exe}}$. Moreover, the total energy consumption of the mobile device is denoted as $E_{k_{n}\left(t\right),2}=E_{n,m,k_{n}\left(t\right)}^{\mathrm{upl}}$.

#### 2.6.3. Case 3: Offloading without Cache

Then, in case 3, the mobile device offloads the inference task to the MEC server without any DNN service cache. The combination of the decision and status satisfies $x_{k_{n}\left(t\right),3}\left(t\right)=x_{m,k_{n}\left(t\right)}^{\mathrm{exe}}\left(t\right)x_{k_{n}\left(t\right),j}^{\mathrm{typ}}\left(1-x_{m,j}^{\mathrm{cac}}\left(t\right)\right)=1$. The total time delay, in this case, can be calculated as follows: $T_{k_{n}\left(t\right),3}=T_{n,m,k_{n}\left(t\right)}^{\mathrm{upl}}+T_{0,m,j}^{\mathrm{lib}}+T_{0,m,j}^{\mathrm{par}}+T_{m,k_{n}\left(t\right)}^{\mathrm{exe}}$. Likewise, the total energy consumption of the mobile device is also represented as $E_{k_{n}\left(t\right),3}=E_{n,m,k_{n}\left(t\right)}^{\mathrm{upl}}$.

#### 2.6.4. Case 4: Local Execution with Fresh Cache

For local execution, in case 4, the mobile device locally executes the inference task with caching service libraries and fresh parameters. The combination of the decision and status satisfies $x_{k_{n}\left(t\right),4}\left(t\right)=\left(1-\sum_{m\in M}x_{m,k_{n}\left(t\right)}^{\mathrm{exe}}\left(t\right)\right)x_{k_{n}\left(t\right),j}^{\mathrm{typ}}x_{n,j}^{\mathrm{cac}}\left(t\right)x_{n,j}^{\mathrm{fre}}\left(t\right)=1$. The total time delay, in this case, can be calculated as follows: $T_{k_{n}\left(t\right),4}=T_{n,k_{n}\left(t\right)}^{\mathrm{exe}}$. In addition, the total energy consumption of the mobile device is denoted as $E_{k_{n}\left(t\right),4}=E_{n,k_{n}\left(t\right)}^{\mathrm{exe}}$.

#### 2.6.5. Case 5: Local Execution with Stale Cache

In case 5, the mobile device locally executes the inference task with caching service libraries and stale parameters. The combination of the decision and status satisfies $x_{k_{n}\left(t\right),5}\left(t\right)=\left(1-\sum_{m\in M}x_{m,k_{n}\left(t\right)}^{\mathrm{exe}}\left(t\right)\right)x_{k_{n}\left(t\right),j}^{\mathrm{typ}}x_{n,j}^{\mathrm{cac}}\left(t\right)\left(1-x_{n,j}^{\mathrm{fre}}\left(t\right)\right)=1$. The total time delay, in this case, can be calculated as $T_{k_{n}\left(t\right),5}=T_{0,n,j}^{\mathrm{par}}+T_{n,k_{n}\left(t\right)}^{\mathrm{exe}}$. Then, the total energy consumption of the mobile device is calculated as $E_{k_{n}\left(t\right),5}=E_{n,k_{n}\left(t\right)}^{\mathrm{exe}}$.

#### 2.6.6. Case 6: Local Execution without Cache

Finally, in case 6, the mobile device locally executes the inference task without any DNN service cache. The combination of the decision and status satisfies $x_{k_{n}\left(t\right),6}\left(t\right)=\left(1-\sum_{m\in M}x_{m,k_{n}\left(t\right)}^{\mathrm{exe}}\left(t\right)\right)x_{k_{n}\left(t\right),j}^{\mathrm{typ}}\left(1-x_{n,j}^{\mathrm{cac}}\left(t\right)\right)=1$. The total time delay, in this case, can be calculated as $T_{k_{n}\left(t\right),6}=T_{0,n,j}^{\mathrm{lib}}+T_{0,n,j}^{\mathrm{par}}+T_{n,k_{n}\left(t\right)}^{\mathrm{exe}}$. Similarly, the total energy consumption of the mobile device is also denoted as $E_{k_{n}\left(t\right),6}=E_{n,k_{n}\left(t\right)}^{\mathrm{exe}}$.

## 3. Problem Formulation

In the AoI-aware caching-assisted asymmetric offloading scenario, the average cost of the mobile device and the total bandwidth between the application server and MEC servers or mobile devices should be considered due to their crucial effectiveness. On the one hand, minimizing the average cost of the mobile device can ensure that the real-time requirements of the generated inference tasks are met and the battery energy is conserved. As the consumed bandwidth of the application server is limited, while it bears other realtime inference tasks, it is required to minimize the total bandwidth consumption between the application server and MEC servers or mobile devices. Accordingly, the average cost of time completion delay is as follows:

(10) $$T^{\mathrm{ave}}=\frac{1}{\left|T||N\right|}\sum_{t\in T}\sum_{n\in N}\left(\sum_{m\in M}\sum_{i=1}^{3}x_{k_{n}\left(t\right),i}\left(t\right)T_{k_{n}\left(t\right),i}+\sum_{i=4}^{6}x_{k_{n}\left(t\right),i}\left(t\right)T_{k_{n}\left(t\right),i}\right),$$

where $x_{k_{n}\left(t\right),i}\left(t\right)$ is defined as $x_{k_{n}\left(t\right),i}\left(t\right)=1$ if $k_{n}\left(t\right)$ is executed via case *i*; otherwise, $x_{k_{n}\left(t\right),i}\left(t\right)=0$, and satisfies that each inference task must be executed via one of the cases at one MEC server or local mobile device: $\sum_{m\in M}\sum_{i=1}^{3}x_{k_{n}\left(t\right),i}\left(t\right)+\sum_{i=4}^{6}x_{k_{n}\left(t\right),i}\left(t\right)=1$. Then, the average cost of energy consumption can be denoted as

(11) $$E^{\mathrm{ave}}=\frac{1}{\left|T||N\right|}\sum_{t\in T}\sum_{n\in N}\left(\sum_{m\in M}\sum_{i=1}^{3}x_{k_{n}\left(t\right),i}\left(t\right)E_{k_{n}\left(t\right),i}+\sum_{i=4}^{6}x_{k_{n}\left(t\right),i}\left(t\right)E_{k_{n}\left(t\right),i}\right),$$

the time average global allocation bandwidth between the application server and MEC servers or mobile devices is denoted as $b_{0}$.

Therefore, we formally formulate the original problem to minimize the time average global allocation bandwidth and the average cost of the mobile device consisting of inference task completion delay and energy consumption:

(12) $$\min_{x_{k_{n}\left(t\right),i}\left(t\right),f_{m}\left(t\right),p_{n,m}\left(t\right)}Z=\xi^{\mathrm{tim}}T^{\mathrm{ave}}+\xi^{\mathrm{ene}}E^{\mathrm{ave}}+\xi^{\mathrm{ban}}b_{0}$$

(13) $$s.t.\;\;T_{k_{n}\left(t\right),i}\leq T_{k_{n}\left(t\right)}^{\max},\forall n\in N,t\in T,i\in \left\{1,\cdots ,6\right\},$$

(14) $$E_{k_{n}\left(t\right),i}\leq E_{k_{n}\left(t\right)}^{\max},\forall n\in N,t\in T,i\in \left\{1,\cdots ,6\right\},$$

(15) $$\sum_{m\in M}\sum_{i=1}^{3}x_{k_{n}\left(t\right),i}\left(t\right)+\sum_{i=4}^{6}x_{k_{n}\left(t\right),i}\left(t\right)=1,$$

(16) $$x_{m,k_{n}\left(t\right)}^{\mathrm{exe}}\left(t\right)\in \left\{0,1\right\},\forall m\in M,n\in N,t\in T,$$

(17) $$x_{m,j}^{\mathrm{cac}}\left(t\right)\in \left\{0,1\right\},\forall m\in M,j\in J,t\in T,$$

(18) $$x_{m,j}^{\mathrm{fre}}\left(t\right)\in \left\{0,1\right\},\forall m\in M,j\in J,t\in T,$$

(19) $$\;\;\sum_{j\in J}x_{m,j}^{\mathrm{cac}}\left(t\right)\left(d_{j}^{\mathrm{lib}}+d_{j}^{\mathrm{par}}\right)\leq d_{m}^{\max},\forall m\in M,j\in J,t\in T,$$

(20) $$\;\;\sum_{j\in J}x_{n,j}^{\mathrm{cac}}\left(t\right)\left(d_{j}^{\mathrm{lib}}+d_{j}^{\mathrm{par}}\right)\leq d_{n}^{\max},\forall n\in N,j\in J,t\in T,$$

(21) $$f_{n}\left(t\right)<f_{m}\left(t\right)\leq f^{\max},\forall m\in M,n\in N,t\in T,$$

(22) $$p_{n,m}\left(t\right)\leq p^{\max},\forall m\in M,n\in N,t\in T,$$

where $x_{k_{n}\left(t\right),i}\left(t\right)$, $f_{m}\left(t\right)$, and $p_{n,m}\left(t\right)$ are optimization variables. $\xi^{\mathrm{ban}}$, $\xi^{\mathrm{tim}}$, and $\xi^{\mathrm{ene}}$ are the given weights of the average global AoI, average time cost, and average energy cost, respectively. (13) and (14) indicate that the inference task completion time delay and consumed energy have upper bounds. According to (15), each inference task has to be executed via (at most) one case at one MEC server or local mobile device. (16)–(18) show that the optimization variables are binary. (19) and (20) constrain the caching capacity limit of heterogeneous services at the MEC server or the mobile device. (21) shows that the computation capability of the MEC server is higher than the mobile device and has a maximum. (22) restricts the upper bound of the uplink transmission power of the mobile device.

$x_{m,k_{n}\left(t\right)}^{\mathrm{exe}}\left(t\right)$, $x_{m,j}^{\mathrm{cac}}\left(t\right)$, and $x_{m,j}^{\mathrm{fre}}\left(t\right)$ are discrete binary integer variables; $p_{n,m}\left(t\right)$ and $f_{m}\left(t\right)$ are continuous variables. The objective functions are not linear to the variables, which are coupled mutually. Therefore, problem (12) is an MINLP problem known as NP-hard. It is difficult to solve the problem within the polynomial time. Combining the practical asymmetric environment, it is more challenging to analyze and propose a solution.

### 3.1. Average Cost Minimization Problem

From the perspective of mobile devices, we first decompose problem (12) into a problem to minimize the average cost of mobile devices:

(23) $$\min_{x_{k_{n}\left(t\right),i}\left(t\right),f_{m}\left(t\right),p_{n,m}\left(t\right)}\xi^{\mathrm{tim}}T^{\mathrm{ave}}+\xi^{\mathrm{ene}}E^{\mathrm{ave}}$$

s.t.(C1)–(C10),

where mobile devices make their decisions based on the weighted sum of time delay and energy consumption.

### 3.2. Bandwidth Consumption Minimization Problem

From the perspective of the application server, the total bandwidth allocated to the requested MEC server or mobile device is constrained when it transmits the requested service data. We secondly decompose problem (12) into a problem minimizing the consumed bandwidth of the application server:

(24) $$\min\xi^{\mathrm{ban}}b_{0}$$

(25) $$s.t.\;b_{0}\leq \frac{1}{\left|T\right|}\sum_{t\in T}b_{0}^{\max},$$

where $b_{0}^{\max}$ is the total allocated bandwidth upper bound of the application server at one timeslot.

### 3.3. Service Fetching Time Minimization Problem

When the application server transmits the service data to the MEC servers or mobile devices, the total transmission time of the responding service data can be minimized based on the total allocated bandwidth. We further formulate problem (26):

(26) $$\min\;T^{\mathrm{fet}}\left(t\right)$$

(27) $$s.t.\;b_{0,m}\left(t\right)+\sum_{n\in N(t)}b_{0,n}\left(t\right)\leq b_{0}\left(t\right),$$

(28) $$x_{k_{n}\left(t\right),2}\left(t\right)+x_{k_{n}\left(t\right),3}\left(t\right)+x_{k_{n}\left(t\right),5}\left(t\right)+x_{k_{n}\left(t\right),6}\left(t\right)=1,$$

(29) $$x_{k_{n}\left(t\right),i}\left(t\right)\in \left\{0,1\right\},i=\left\{2,3,5,6\right\},$$

where $T^{\mathrm{fet}}\left(t\right)$ is the total transmission time of the responding service data. (27) indicates that the total allocated bandwidth has an upper bound, (28) and (29) limit the combination decisions.

Here, we clarify the connections among these three subproblems and how they can work together to reach the optimal solution for problem (12). Problem (12) jointly minimizes the cost of mobile devices and the global allocation bandwidth. Firstly, problem (23) minimizes the mobile device cost, including the time delay and energy consumption. Secondly, problem (24) minimizes the time average allocation bandwidth from a global perspective. Thirdly, problem (26) further minimizes the responding service transmission time after making the offloading decision based on the solution of the problem (23).

## 4. Solution

In this section, we propose three modules to, respectively, solve the subproblems in the last section. In particular, the LMKO module can minimize the average cost of mobile devices. To minimize the consumed bandwidth of the application server, we devise the LLUC module. Moreover, the KCDF module minimizes the total transmission time of the responding service data.

### 4.1. Method of Lagrange Multipliers with the KKT Condition-Based Offloading Module (LMKO)

To minimize the average cost of mobile devices, we transform problem (23) into a problem of tractable form and further leverage convex optimization to solve it. According to constraint (13), the time delay of each case cannot exceed the inference task completion tolerance deadline. Hence, we set the time delay of the case with most of the procedures to the maximum tolerance time to reduce the number of optimization variables: $T_{k_{n}\left(t\right),3}=T_{k_{n}\left(t\right)}^{\max}$. For succinct expression, we define notations *A* and *B* as

(30) $$A=T_{k_{n}\left(t\right)}^{\max}-T_{0,m,j}^{\mathrm{lib}}-T_{0,m,j}^{\mathrm{par}},$$

(31) $$B=\frac{\sigma^{2}+I_{n,m}\left(t\right)}{h_{n,m}\left(t\right)}.$$

Then, $f_{m}\left(t\right)$ and $p_{n,m}\left(t\right)$ can be transform into the function values of $g_{1}\left(T_{m,k_{n}\left(t\right)}^{\mathrm{exe}}\left(t\right)\right)$ and $g_{2}\left(T_{m,k_{n}\left(t\right)}^{\mathrm{exe}}\left(t\right)\right)$, respectively:

(32) $$f_{m}\left(t\right)=g_{1}\left(T_{m,k_{n}\left(t\right)}^{\mathrm{exe}}\left(t\right)\right)=\frac{c_{k_{n}\left(t\right)}}{T_{m,k_{n}\left(t\right)}^{\mathrm{exe}}\left(t\right)},$$

(33) $$\;\;\;\;p_{n,m}\left(t\right)=g_{2}\left(T_{m,k_{n}\left(t\right)}^{\mathrm{exe}}\left(t\right)\right)=\left(2^{\frac{d_{k_{n}\left(t\right)}}{b_{n,m}\left(t\right)\left(A-T_{m,k_{n}\left(t\right)}^{\mathrm{exe}}\right)}}-1\right)B.$$

Moreover, the cost of the objective function in problem (23) can be calculated as

(34) $$\begin{array}{cc} Z_{1} & =\xi^{\mathrm{tim}}\frac{1}{\left|T||N\right|}\sum_{t\in T}\sum_{n\in N}(\sum_{m\in M}(x_{k_{n}\left(t\right),1}\left(t\right)A\\ & +x_{k_{n}\left(t\right),2}\left(t\right)\left(T_{k_{n}\left(t\right)}^{\max}-T_{0,m,j}^{\mathrm{lib}}\right)\\ & +x_{k_{n}\left(t\right),3}\left(t\right)T_{k_{n}\left(t\right)}^{\max})+\sum_{i=4}^{6}x_{k_{n}\left(t\right),i}\left(t\right)T_{k_{n}\left(t\right),i})\;\;\;\;\\ & +\xi^{\mathrm{ene}}\frac{1}{\left|T||N\right|}\sum_{t\in T}\sum_{n\in N}(\sum_{m\in M}\sum_{i=1}^{3}x_{k_{n}\left(t\right),i}\left(t\right)\\ & \times g_{2}\left(T_{m,k_{n}\left(t\right)}^{\mathrm{exe}}\left(t\right)\right)\left(A-T_{m,k_{n}\left(t\right)}^{\mathrm{exe}}\left(t\right)\right)\\ & +\sum_{i=4}^{6}x_{k_{n}\left(t\right),i}\left(t\right)E_{k_{n}\left(t\right),i}). \end{array}$$

Therefore, problem (23) can be further transformed into:

(35) $$\min_{T_{m,k_{n}\left(t\right)}^{\mathrm{exe}}\left(t\right),x_{k_{n}\left(t\right),i}\left(t\right)}Z_{1}$$

(36) $$s.t.\;g_{1}\left(T_{m,k_{n}\left(t\right)}^{\mathrm{exe}}\left(t\right)\right)\leq f^{\max},$$

(37) $$g_{2}\left(T_{m,k_{n}\left(t\right)}^{\mathrm{exe}}\left(t\right)\right)\leq p^{\max},$$

(38) $$x_{k_{n}\left(t\right),i}\left(t\right)\in \left[0,1\right],$$

where $T_{m,k_{n}\left(t\right)}^{\mathrm{exe}}\left(t\right)$ and $x_{k_{n}\left(t\right),i}\left(t\right)$ are optimization variables. Constraint (36) reflects the computation capability limit and $T_{m,k_{n}\left(t\right)}^{\mathrm{exe}}\left(t\right)$. (37) constrains the relationship between the maximum power and $T_{m,k_{n}\left(t\right)}^{\mathrm{exe}}\left(t\right)$. (38) indicates that the decision combination is relaxed to be continuous.

Subsequently, we leverage the method using Lagrange multipliers with KKT conditions [25] to solve problem (35). Before this, we prove that the problem (35) is convex.

Now, we define another function of $T_{m,k_{n}\left(t\right)}^{\mathrm{exe}}\left(t\right)$ as follows:

(39) $$g_{3}\left(T_{m,k_{n}\left(t\right)}^{\mathrm{exe}}\left(t\right)\right)=g_{2}\left(T_{m,k_{n}\left(t\right)}^{\mathrm{exe}}\left(t\right)\right)\left(A-T_{m,k_{n}\left(t\right)}^{\mathrm{exe}}\left(t\right)\right),$$

and take the second partial derivative of $g_{3}\left(T_{m,k_{n}\left(t\right)}^{\mathrm{exe}}\left(t\right)\right)$ with respect to $T_{m,k_{n}\left(t\right)}^{\mathrm{exe}}\left(t\right)$:

(40) $$\frac{\partial^{2}g_{3}}{\partial {T_{m,k_{n}\left(t\right)}^{\mathrm{exe}}}^{2}}=\frac{{\ln2}^{2}{d_{k_{n}\left(t\right)}}^{2}B2^{\frac{d_{k_{n}\left(t\right)}}{b_{n,m}\left(t\right)\left(A-T_{m,k_{n}\left(t\right)}^{\mathrm{exe}}\left(t\right)\right)}}}{{b_{n,m}\left(t\right)}^{2}{\left(A-T_{m,k_{n}\left(t\right)}^{\mathrm{exe}}\left(t\right)\right)}^{3}}.$$

All of the terms in (40) are positive, $\frac{\partial^{2}g_{3}}{\partial {T_{m,k_{n}\left(t\right)}^{\mathrm{exe}}}^{2}}>0$, and $g_{3}\left(T_{m,k_{n}\left(t\right)}^{\mathrm{exe}}\left(t\right)\right)$ is a convex function. Similarly, $g_{3}\left(x_{k_{n}\left(t\right),i}\left(t\right)T_{m,k_{n}\left(t\right)}^{\mathrm{exe}}\left(t\right)\right)$ is a convex function. Furthermore, we define a perspective function of $g_{3}\left(x_{k_{n}\left(t\right),i}\left(t\right)T_{m,k_{n}\left(t\right)}^{\mathrm{exe}}\left(t\right)\right)$ as

(41) $$\begin{array}{cc} \; & g_{4}\left(x_{k_{n}\left(t\right),i}\left(t\right)T_{m,k_{n}\left(t\right)}^{\mathrm{exe}}\left(t\right),x_{k_{n}\left(t\right),i}\left(t\right)\right)\\ = & x_{k_{n}\left(t\right),i}\left(t\right)g_{3}\left(\frac{x_{k_{n}\left(t\right),i}\left(t\right)T_{m,k_{n}\left(t\right)}^{\mathrm{exe}}\left(t\right)}{x_{k_{n}\left(t\right),i}\left(t\right)}\right)\\ = & x_{k_{n}\left(t\right),i}\left(t\right)g_{2}\left(T_{m,k_{n}\left(t\right)}^{\mathrm{exe}}\left(t\right)\right)\left(A-T_{m,k_{n}\left(t\right)}^{\mathrm{exe}}\left(t\right)\right). \end{array}$$

According to (41), $g_{4}\left(x_{k_{n}\left(t\right),i}\left(t\right)T_{m,k_{n}\left(t\right)}^{\mathrm{exe}}\left(t\right),x_{k_{n}\left(t\right),i}\left(t\right)\right)$ is convex so that the objective function of the problem (35) is a convex function. Then, the second partial derivatives of $g_{1}\left(T_{m,k_{n}\left(t\right)}^{\mathrm{exe}}\left(t\right)\right)$ and $g_{2}\left(T_{m,k_{n}\left(t\right)}^{\mathrm{exe}}\left(t\right)\right)$ with respect to $T_{m,k_{n}\left(t\right)}^{\mathrm{exe}}\left(t\right)$ are, respectively, calculated as (42) and (43):

(42) $$\frac{\partial^{2}g_{1}}{\partial {T_{m,k_{n}\left(t\right)}^{\mathrm{exe}}}^{2}}=\frac{2C_{k_{n}\left(t\right)}}{{T_{m,k_{n}\left(t\right)}^{\mathrm{exe}}\left(t\right)}^{3}},$$

(43) $$\begin{array}{cc} \frac{\partial^{2}g_{2}}{\partial {T_{m,k_{n}\left(t\right)}^{\mathrm{exe}}}^{2}}= & \frac{\ln2d_{k_{n}\left(t\right)}B2^{\frac{d_{k_{n}\left(t\right)}}{b_{n,m}\left(t\right)\left(A-T_{m,k_{n}\left(t\right)}^{\mathrm{exe}}\left(t\right)\right)}}}{b_{n,m}\left(t\right){\left(A-T_{m,k_{n}\left(t\right)}^{\mathrm{exe}}\left(t\right)\right)}^{3}}\\ & \times (\frac{\ln2d_{k_{n}\left(t\right)}}{b_{n,m}\left(t\right)\left(A-T_{m,k_{n}\left(t\right)}^{\mathrm{exe}}\left(t\right)\right)}+2). \end{array}$$

All terms in (42) and (43) are positive, $\frac{\partial^{2}g_{1}}{\partial {T_{m,k_{n}\left(t\right)}^{\mathrm{exe}}}^{2}}>0$, and $\frac{\partial^{2}g_{2}}{\partial {T_{m,k_{n}\left(t\right)}^{\mathrm{exe}}}^{2}}>0$. Thus, constraint (36) and (37) are convex with $T_{m,k_{n}\left(t\right)}^{\mathrm{exe}}\left(t\right)$. The feasible region of the Problem (35) is a convex set. We can derive that the Problem (35) is convex. In addition, if $p^{\max}$ and $f^{\max}$ are high enough, we can find a feasible solution to making all of the constraints slack, hence satisfying the Slater condition. A convex problem that satisfies the Slater condition is sufficient for the problem and its dual problem to be strong. In other words, they have zero dual gap and their optimal solutions are equal.

Next, we define the Lagrangian relaxation function of the problem (35) as

(44) $$\begin{array}{cc} \; & \;\;\;\;\;\;\;\;L(T_{m,k_{n}\left(t\right)}^{\mathrm{exe}}\left(t\right),x_{k_{n}\left(t\right),i}\left(t\right),\lambda_{m,k_{n}\left(t\right),1},\lambda_{m,k_{n}\left(t\right),2})\\ \;\;\;\;\;\;\;\;= & \;\;\;\;\;\;\;\;C\;+\;\sum_{t\in T}\sum_{n\in N}\sum_{m\in M}(\lambda_{m,k_{n}\left(t\right),1}\left(g_{1}\left(T_{m,k_{n}\left(t\right)}^{\mathrm{exe}}\left(t\right)\right)\;-\;f^{\max}\right)\\ & \;\;\;\;\;\;\;\;+\lambda_{m,k_{n}\left(t\right),2}\left(g_{2}\left(T_{m,k_{n}\left(t\right)}^{\mathrm{exe}}\left(t\right)\right)-p^{\max}\right)), \end{array}$$

where $\lambda_{m,k_{n}\left(t\right),1}$ and $\lambda_{m,k_{n}\left(t\right),2}$ are the Lagrangian multipliers. The Lagrangian relaxation function relaxes the constraints of the Problem (35). Here, we formally transform problem (35) into its dual problem:

(45) $$\max_{\lambda_{m,k_{n}\left(t\right),1},\lambda_{m,k_{n}\left(t\right),2}}\;\min_{T_{m,k_{n}\left(t\right)}^{\mathrm{exe}}\left(t\right),x_{k_{n}\left(t\right),i}\left(t\right)}L$$

(46) $$s.t.\;\lambda_{m,k_{n}\left(t\right),1}\geq 0,\;\lambda_{m,k_{n}\left(t\right),2}\geq 0,$$

where we first fix $\lambda_{m,k_{n}\left(t\right),1}$, $\lambda_{m,k_{n}\left(t\right),2}$ and minimize *L* to obtain the infimum, then fix $T_{m,k_{n}\left(t\right)}^{\mathrm{exe}}\left(t\right)$, $x_{k_{n}\left(t\right),i}\left(t\right)$ and maximize the infimum. (46) indicates that the Lagrangian multipliers are positive.

We further detail the KKT condition of the problem (45):

(47) $$\;\;\;\;\;\;\begin{array}{cc} \frac{\partial L}{\partial T_{m,k_{n}\left(t\right)}^{\mathrm{exe}}}= & \frac{\xi^{\mathrm{ene}}}{\left|T||N\right|}\sum_{t\in T}\sum_{n\in N}\sum_{m\in M}x_{k_{n}\left(t\right),i}\left(t\right)\frac{\partial g_{3}}{\partial T_{m,k_{n}\left(t\right)}^{\mathrm{exe}}}\\ & +\sum_{t\in T}\sum_{n\in N}\sum_{m\in M}(\lambda_{m,k_{n}\left(t\right),1}\frac{\partial g_{1}}{\partial T_{m,k_{n}\left(t\right)}^{\mathrm{exe}}}\\ & +\lambda_{m,k_{n}\left(t\right),2}\frac{\partial g_{2}}{\partial T_{m,k_{n}\left(t\right)}^{\mathrm{exe}}})=0, \end{array}$$

(48) $$\lambda_{m,k_{n}\left(t\right),1}\left(g_{1}\left(T_{m,k_{n}\left(t\right)}^{\mathrm{exe}}\left(t\right)\right)-f^{\max}\right)=0,$$

(49) $$\lambda_{m,k_{n}\left(t\right),2}\left(g_{2}\left(T_{m,k_{n}\left(t\right)}^{\mathrm{exe}}\left(t\right)\right)-p^{\max}\right)=0,$$

(50) $$g_{1}\left(T_{m,k_{n}\left(t\right)}^{\mathrm{exe}}\left(t\right)\right)\leq f^{\max},$$

(51) $$g_{2}\left(T_{m,k_{n}\left(t\right)}^{\mathrm{exe}}\left(t\right)\right)\leq p^{\max},$$

(52) $$\lambda_{m,k_{n}\left(t\right),1}\geq 0,\;\lambda_{m,k_{n}\left(t\right),2}\geq 0,$$

where

(53) $$\frac{\partial g_{3}}{\partial T_{m,k_{n}\left(t\right)}^{\mathrm{exe}}}=B\left(\frac{\ln2d_{k_{n}\left(t\right)}2^{\frac{d_{k_{n}\left(t\right)}}{b_{n,m}\left(t\right)\left(A-T_{m,k_{n}\left(t\right)}^{\mathrm{exe}}\right)}}}{b_{n,m}\left(t\right)\left(A-T_{m,k_{n}\left(t\right)}^{\mathrm{exe}}\right)}-2^{\frac{d_{k_{n}\left(t\right)}}{b_{n,m}\left(t\right)\left(A-T_{m,k_{n}\left(t\right)}^{\mathrm{exe}}\right)}}+1\right),$$

(54) $$\frac{\partial g_{1}}{\partial T_{m,k_{n}\left(t\right)}^{\mathrm{exe}}}=-\frac{c_{k_{n}\left(t\right)}}{{T_{m,k_{n}\left(t\right)}^{\mathrm{exe}}}^{2}},$$

and

(55) $$\frac{\partial g_{2}}{\partial T_{m,k_{n}\left(t\right)}^{\mathrm{exe}}}=\frac{B\ln2d_{k_{n}\left(t\right)}2^{\frac{d_{k_{n}\left(t\right)}}{b_{n,m}\left(t\right)\left(A-T_{m,k_{n}\left(t\right)}^{\mathrm{exe}}\right)}}}{b_{n,m}\left(t\right){\left(A-T_{m,k_{n}\left(t\right)}^{\mathrm{exe}}\right)}^{2}}.$$

In KKT conditions, (47) is the dual stationarity condition. (48) and (49) are the complementary slackness conditions. (50) and (51) are the primal feasibility conditions while (52) is the dual feasibility condition.

Since (47) is a transcendental equation, we derive the solution by the Newton iteration method:

(56) $${T_{m,k_{n}\left(t\right)}^{\mathrm{exe}}}^{\prime }=T_{m,k_{n}\left(t\right)}^{\mathrm{exe}}-\frac{\frac{\partial L}{\partial T_{m,k_{n}\left(t\right)}^{\mathrm{exe}}}}{\frac{\partial^{2}L}{\partial {T_{m,k_{n}\left(t\right)}^{\mathrm{exe}}}^{2}}},$$

where

(57) $$\begin{array}{cc} \frac{\partial^{2}L}{\partial {T_{m,k_{n}\left(t\right)}^{\mathrm{exe}}}^{2}}= & \frac{\xi^{\mathrm{ene}}}{\left|T||N\right|}\sum_{t\in T}\sum_{n\in N}\sum_{m\in M}x_{k_{n}\left(t\right),i}\left(t\right)\frac{\partial^{2}g_{3}}{\partial {T_{m,k_{n}\left(t\right)}^{\mathrm{exe}}}^{2}}\\ & +\sum_{t\in T}\sum_{n\in N}\sum_{m\in M}\left(\lambda_{m,k_{n}\left(t\right),1}\frac{\partial^{2}g_{1}}{\partial {T_{m,k_{n}\left(t\right)}^{\mathrm{exe}}}^{2}}+\lambda_{m,k_{n}\left(t\right),2}\frac{\partial^{2}g_{2}}{\partial {T_{m,k_{n}\left(t\right)}^{\mathrm{exe}}}^{2}}\right)=0. \end{array}$$

After iterations of the Newton method, we obtain the optimal solution ${T_{m,k_{n}\left(t\right)}^{\mathrm{exe}}}^{*}$. Then, the optimal resource allocation ${f_{m}\left(t\right)}^{*}$ and ${p_{n,m}\left(t\right)}^{*}$ can be calculated according to (32) and (33), respectively.

Moreover, we leverage the subgradient method to update the Lagrangian multipliers:

(58) $$\;\;\;\;\begin{array}{cc} {\lambda_{m,k_{n}\left(t\right),1}}^{\prime } & =\max\{\lambda_{m,k_{n}\left(t\right),1}\\ & +\alpha_{m,k_{n}\left(t\right),1}\left(g_{1}\left(T_{m,k_{n}\left(t\right)}^{\mathrm{exe}}\left(t\right)\right)-f^{\max}\right),0\}, \end{array}$$

(59) $$\;\;\;\;\begin{array}{cc} {\lambda_{m,k_{n}\left(t\right),2}}^{\prime } & =\max\{\lambda_{m,k_{n}\left(t\right),2}\\ & +\alpha_{m,k_{n}\left(t\right),2}\left(g_{2}\left(T_{m,k_{n}\left(t\right)}^{\mathrm{exe}}\left(t\right)\right)-p^{\max}\right),0\}, \end{array}$$

where $\alpha_{m,k_{n}\left(t\right),1}$ and $\alpha_{m,k_{n}\left(t\right),2}$ are the diminishing step size, respectively.

Since problem (45) is convex with respect to the optimization variable, the update iteration can converge to the optimal solution, satisfying the following conditions: $\sum_{\tau^{\mathrm{sub}}=1}^{\infty }\alpha_{m,k_{n}\left(t\right),1}\left(\tau^{\mathrm{sub}}\right)=\infty$, $\sum_{\tau^{\mathrm{sub}}=1}^{\infty }\alpha_{m,k_{n}\left(t\right),2}\left(\tau^{\mathrm{sub}}\right)=\infty$, $\sum_{\tau^{\mathrm{sub}}=1}^{\infty }{\alpha_{m,k_{n}\left(t\right),1}\left(\tau^{\mathrm{sub}}\right)}^{2}<\infty$, and $\sum_{\tau^{\mathrm{sub}}=1}^{\infty }{\alpha_{m,k_{n}\left(t\right),2}\left(\tau^{\mathrm{sub}}\right)}^{2}<\infty$, where $\tau^{\mathrm{sub}}$ is the iteration index. There is proof in [26].

Based on the given service caching placement decision and service parameter freshness status, we can calculate the cost of local execution as follows:

(60) $$\;\;\;\;C_{k_{n}\left(t\right)}^{\mathrm{loc}}=x_{k_{n}\left(t\right),i}\left(t\right)\left(\xi^{\mathrm{tim}}T_{k_{n}\left(t\right)}^{*}+\xi^{\mathrm{ene}}E_{k_{n}\left(t\right)}^{*}\right),i\in \left\{1,2,3\right\},\;\;$$

the offloading cost of the MEC server *m* is calculated as

(61) $$\;\;\;\;\;C_{m,k_{n}\left(t\right)}^{\mathrm{off}}\;=\;x_{k_{n}\left(t\right),i}\left(t\right)\left(\xi^{\mathrm{tim}}T_{k_{n}\left(t\right)}^{*}\;+\;\xi^{\mathrm{ene}}E_{k_{n}\left(t\right)}^{*}\right),i\in \left\{4,5,6\right\},\;\;$$

and the minimum offloading cost among all the MEC servers is calculated as

(62) $$C_{m^{*},k_{n}\left(t\right)}^{\mathrm{off}}=\min_{m\in M}C_{m,k_{n}\left(t\right)}^{\mathrm{off}}$$

where $T_{k_{n}\left(t\right)}^{*}$ and $E_{k_{n}\left(t\right)}^{*}$ are calculated according to ${f_{m}\left(t\right)}^{*}$ and ${p_{n,m}\left(t\right)}^{*}$. The offloading decision can be further derived. If $C_{k_{n}\left(t\right)}^{\mathrm{loc}}<C_{m^{*},k_{n}\left(t\right)}^{\mathrm{off}}$, $x_{m^{*},k_{n}\left(t\right)}^{\mathrm{exe}}\left(t\right)=1$, and $x_{m,k_{n}\left(t\right)}^{\mathrm{exe}}\left(t\right)=0,\;\forall m\in M\backslash m^{*}$, the inference task is offloaded to the MEC server $m^{*}$; otherwise, $x_{m,k_{n}\left(t\right)}^{\mathrm{exe}}\left(t\right)=0,\;\forall m\in M$, and the inference task is executed locally.

The pseudo-code of the LMKO is shown in Algorithm 1. The complexity of LMKO is $O(|M|\left(\tau^{\mathrm{new}}+\tau^{\mathrm{sub}}\right))+|M|)$, where $\tau^{\mathrm{new}}$ and $\tau^{\mathrm{sub}}$ are the iteration numbers of the Newton method and the subgradient method, respectively.

### 4.2. Lyapunov Optimization-Based Learning and Update Control Module (LLUC)

Since the application server also bears other applications, its bandwidth resources are limited and need to be economized. In this subsection, we minimize the bandwidth consumption of the application server from the perspective of a global view while minimizing the service fetching time to accelerate the inference task processing.

The inference tasks are generated randomly, and the execution request in the offloading style or local style is a random event for the MEC server and mobile device. If there is no caching service or fresh service parameters, they call for the application server to fetch the service. Therefore, the fetching request is also random in terms of the application server, which has no a priori distribution. We regard the total requested service data size waiting for transmission as a queue, and leverage the Lyapunov optimization to solve the problem of stabilizing a randomly arriving queue system.

The application server transmits the requested service data as soon as possible to decrease the fetching time. At timeslot *t*, the total requested service data size can be defined as the enqueued rate:

(63) $$\begin{array}{cc} d^{\mathrm{enq}}\left(t\right)= & \sum_{n\in N}(\sum_{m\in M}(x_{k_{n}\left(t\right),2}\left(t\right)d_{j}^{\mathrm{par}}\\ & +x_{k_{n}\left(t\right),3}\left(t\right)\left(d_{j}^{\mathrm{lib}}+d_{j}^{\mathrm{par}}\right))+x_{k_{n}\left(t\right),5}\left(t\right)d_{j}^{\mathrm{par}}+x_{k_{n}\left(t\right),6}\left(t\right)\left(d_{j}^{\mathrm{lib}}+d_{j}^{\mathrm{par}}\right)). \end{array}$$

Moreover, let $d^{\mathrm{deq}}$ be the dequeued rate, which is the total size of the service transmitted from the application server to the requesting MEC servers or mobile devices. Furthermore, the backlog of the queue can be defined as $Q\left(t+1\right)=\max\{Q\left(t\right)+d^{\mathrm{enq}}\left(t\right)-d^{\mathrm{deq}}\left(t\right),0\}$, where the enqueued rate and dequeued rate can affect the queue backlog of the next timeslot. Then, we define the quadratic Lyapunov function as $Y\left(t\right)=\frac{{Q(t)}^{2}}{2}$, and the Lyapunov drift can be denoted as ΔY(t)=Y(t+1)−Y(t). In addition, we define the penalty function of the Lyapunov optimization, which equals the total allocated bandwidth consumption for transmitting the requested service data at timeslot *t*: $b_{0}\left(t\right)=\beta d^{\mathrm{deq}}\left(t\right)$, where β is the simplified transformation coefficient.

We formally transform problem (24) into the Lyapunov optimization problem:

(64) $$\min_{d^{\mathrm{deq}}\left(t\right)}Z_{2}=\Delta Y\left(t\right)+V\left(t\right)b_{0}\left(t\right)$$

(65) $$s.t.\;\lim_{t\to \infty }\frac{Q(t)}{t}=0,$$

(66) $$b_{0}\left(t\right)\leq b_{0}^{\max},$$

where V(t) is the adaptive weight of the penalty, and (65) is the stable condition of the queue system, and $b_{0}^{\max}$ in (66) is the maximum of the total available bandwidth between the application server and all requesting MEC servers or mobile devices.
**Algorithm 1** Method of Lagrange multipliers with the KKT condition-based offloading module (LMKO).**Require**:time cost $T_{k_{n}\left(t\right),i}$, energy cost $E_{k_{n}\left(t\right),i}$, time weight $\xi^{\mathrm{tim}}$, delay weight $\xi^{\mathrm{ene}}$, maximum tolerance time $T_{k_{n}\left(t\right)}^{\max}$, maximum computation capability $f^{\max}$, maximum power $p^{\max}$, maximum Newton iteration $\tau^{\mathrm{new}}$, subgradient threshold $\epsilon^{\mathrm{sub}}$**Ensure**:offloading decision $x_{m,k_{n}\left(t\right)}^{\mathrm{exe}}\left(t\right)$1:**for**t=1 to |T| **do**2:   **for** t=1 to |N| **do**3:     **for** t=1 to |M| **do**4:        **for** t=1 to $\tau^{\mathrm{new}}$ **do**5:          Calculate ${T_{m,k_{n}\left(t\right)}^{\mathrm{exe}}}^{\prime }$ based on the Newton iteration method according to (56).6:        **end for**7:        Obtain ${T_{m,k_{n}\left(t\right)}^{\mathrm{exe}}}^{*}$.8:        **repeat**9:          Update ${\lambda_{m,k_{n}\left(t\right),1}}^{\prime }$ and ${\lambda_{m,k_{n}\left(t\right),2}}^{\prime }$ based on the subgradient method according to (58) and (59), respectively.10:        **until** ${\lambda_{m,k_{n}\left(t\right),1}}^{\prime }-\lambda_{m,k_{n}\left(t\right),1}\leq \epsilon^{\mathrm{sub}}$ and ${\lambda_{m,k_{n}\left(t\right),2}}^{\prime }-\lambda_{m,k_{n}\left(t\right),2}\leq \epsilon^{\mathrm{sub}}$11:        Calculate ${f_{m}\left(t\right)}^{*}$, ${p_{n,m}\left(t\right)}^{*}$, and $C_{m,k_{n}\left(t\right)}^{\mathrm{off}}$ according to (32), (33), and (61), respectively.12:     **end for**13:     Calculate $C_{k_{n}\left(t\right)}^{\mathrm{loc}}$ and $C_{m^{*},k_{n}\left(t\right)}^{\mathrm{off}}$ according to (60) and (62), respectively.14:     **if** $C_{m^{*},k_{n}\left(t\right)}^{\mathrm{off}}>C_{k_{n}\left(t\right)}^{\mathrm{loc}}$ **then**15:        $x_{m^{*},k_{n}\left(t\right)}^{\mathrm{exe}}\left(t\right)=1$.16:        $x_{m,k_{n}\left(t\right)}^{\mathrm{exe}}\left(t\right)=0,\;\forall m\in M\backslash m^{*}$.17:     **else**18:        $x_{m,k_{n}\left(t\right)}^{\mathrm{exe}}\left(t\right)=0,\;\forall m\in M$.19:     **end if**20:   **end for**21:**end for**22:**return** $x_{m,k_{n}\left(t\right)}^{\mathrm{exe}}\left(t\right)$

**Theorem 1**  ([27]). *Assuming there are constants D≥0, $\epsilon^{\mathrm{que}}>0$, $V^{\max}\geq 0$, $b_{0}^{\max}>0$, such that for all t and all possible variables Q(t), the Lyapunov drift-plus-penalty condition holds that:*

(67) $$E\left[\Delta Y\left(t\right)+V\left(t\right)b_{0}\left(t\right)|Q\left(t\right)\right]\leq D-\epsilon^{\mathrm{que}}Q\left(t\right)+V^{\max}b_{0}^{\max},$$

*where $E[\frac{1}{2}{\left(d^{\mathrm{enq}}\left(t\right)-d^{\mathrm{deq}}\left(t\right)\right)}^{2}|Q\left(t\right)]\leq D$ indicates that the difference between the enqueued rate and the dequeued rate has an upper bound, $E\left[d^{\mathrm{enq}}\left(t\right)-d^{\mathrm{deq}}\left(t\right)|Q\left(t\right)\right]\leq -\epsilon^{\mathrm{que}}$ indicates that the queue is controlled, $V^{\max}$ is the maximum of V(t) over time, and $b_{0}^{\max}$ is the maximum of $b_{0}\left(t\right)$ mentioned above. For all t>0, the time average queue backlog and the time average bandwidth satisfy the following:*

(68) $$\frac{1}{\left|T\right|}\sum_{t=1}^{\left|T\right|}E\left[Q\left(t\right)\right]\leq \frac{D+V^{\max}\left(b_{0}^{\max}-b_{0}^{\min}\right)}{\epsilon^{\mathrm{que}}}+\frac{E[L(1)]}{\left|T\right|\epsilon^{\mathrm{que}}},$$

(69) $$\frac{1}{\left|T\right|}\sum_{t=1}^{\left|T\right|}E\left[b_{0}\left(t\right)\right]\leq \frac{D}{V^{\max}}+b_{0}^{\max}+\frac{E[L(1)]}{\left|T\right|V^{\max}},$$

*where $b_{0}^{\min}$ is the minimum of $b_{0}\left(t\right)$.*

Theorem 1 explains that when the Lyapunov drift-plus-penalty condition is met, the average queue backlog is at most $O(V^{\max})$ complexity, and the average bandwidth is at most $O(\frac{1}{V^{\max}})$ above the maximum bandwidth. Hence, we find that there is a trade-off between the queue backlog and the bandwidth penalty, which is tuned by V(t).

In addition, since $\Delta Y\left(t\right)+V\left(t\right)b_{0}\left(t\right)\leq \frac{{\left(d^{\mathrm{enq}}\left(t\right)-d^{\mathrm{deq}}\left(t\right)\right)}^{2}}{2}+Q\left(t\right)\left(d^{\mathrm{enq}}\left(t\right)-d^{\mathrm{deq}}\left(t\right)\right)+V\left(t\right)\beta d^{\mathrm{deq}}\left(t\right)$, we can derive the optimal controlled dequeued rate by taking the derivative with respect to $d^{\mathrm{deq}}\left(t\right)$ and further setting it to 0:

(70) $$d^{\mathrm{deq},*}\left(t\right)=Q\left(t\right)-V\left(t\right)\beta +d^{\mathrm{enq}}\left(t\right).$$

To improve the Lyapunov optimization, we first design an adaptive learning penalty weight method to adaptively adjust to V(t):

(71) $$V\left(t\right)=\frac{V(1)}{e^{\zeta \phi (t)}},$$

where ζ is the learning rate of penalty weight, $\phi \left(t\right)=\frac{1}{\left|N\right|}\sum_{n\in N(t)}𝟙\left\{T_{k_{n}\left(t\right)}>T_{k_{n}\left(t\right)}^{\max}\right\}$ represents the ratio of the missing tolerance time inference task number, and 𝟙 is an indicator function. The emphasis on the bandwidth penalty is lowered as the ratio of the overtime inference tasks increases. When the ratio is alleviated, the weight of the bandwidth is set to be higher.

Secondly, since the transmission data sizes among all the requested MEC servers or mobile devices are distinct, e.g., some request the service libraries and parameters while others only request the parameters, the application server can preferentially respond to request only to the service parameters to decrease the consumption of the bandwidth when the weight of the bandwidth penalty is high. Therefore, we devise a dequeued rate update mechanism. When $V\left(t\right)>\epsilon^{\mathrm{thr}}$ where $\epsilon^{\mathrm{thr}}$ is a given threshold of the penalty weight, the enqueued rate can be updated to:

(72) $$\;\;\;\;\;d^{\mathrm{deq},\prime }\left(t\right)\;=\;\sum_{m\in M}\sum_{n\in N}\sum_{j\in J}\left(x_{k_{n}\left(t\right),2}\left(t\right)d_{j}^{\mathrm{par}}+x_{k_{n}\left(t\right),5}\left(t\right)d_{j}^{\mathrm{par}}\right),$$

(73) $$\begin{array}{cc} \;\;\;\;\;\;\;\;\;d^{\mathrm{deq},\prime }\left(t+1\right)\;=\;\;\;\; & d^{\mathrm{deq},*}\left(t+1\right)+\sum_{m\in M}\sum_{n\in N}\sum_{j\in J}(x_{k_{n}\left(t\right),3}\left(t\right)\\ & \times \left(d_{j}^{\mathrm{lib}}+d_{j}^{\mathrm{par}}\right)\;+\;x_{k_{n}\left(t\right),6}\left(t\right)\left(d_{j}^{\mathrm{lib}}+d_{j}^{\mathrm{par}}\right)), \end{array}$$

where the request transmission data sizes of cases 3 and 6 are assigned to timeslot t+1 to alleviate the bandwidth penalty at the current timeslot *t*.

Thirdly, from the perspective of the service parameters with few timeslots until the next training, if the application server directly send the part of data, it can be requested again soon due to its stale service parameters. We further propose a freshness-aware transmitting method to reduce the service-requested frequency; the service parameters that will be trained soon are arranged to be transmitted at the end of their training. If the time condition satisfies $\left(\left(t-t_{j}\right)\mathrm{mod}T_{j}^{\mathrm{int}}\right)>\eta T_{j}^{\mathrm{int}}$, where η is the given proportion of the training round and mod is an operator of taking the remainder, the dequeued rate is arranged as follows:

(74) $$\begin{array}{cc} \;\;\;\;\;\;\;d^{\mathrm{deq},\prime }\left(t\right)= & d^{\mathrm{deq},*}\left(t\right)-\sum_{m\in M}\sum_{n\in N}\sum_{\bar{j}\in J\backslash j}(x_{k_{n}\left(t\right),2}\left(t\right)d_{\bar{j}}^{\mathrm{par}}\\ & +x_{k_{n}\left(t\right),3}\left(t\right)\left(d_{\bar{j}}^{\mathrm{lib}}+d_{\bar{j}}^{\mathrm{par}}\right)+x_{k_{n}\left(t\right),5}\left(t\right)d_{\bar{j}}^{\mathrm{par}}\\ & +x_{k_{n}\left(t\right),6}\left(t\right)\left(d_{\bar{j}}^{\mathrm{lib}}+d_{\bar{j}}^{\mathrm{par}}\right)), \end{array}$$

(75) $$\begin{array}{cc} \; & d^{\mathrm{deq},\prime }\left(t+T_{j}^{\mathrm{int}}-\left(\left(t-t_{j}\right)\mathrm{mod}T_{j}^{\mathrm{int}}\right)\right)\\ = & d^{\mathrm{deq},*}\left(t+T_{j}^{\mathrm{int}}-\left(\left(t-t_{j}\right)\mathrm{mod}T_{j}^{\mathrm{int}}\right)\right)\\ + & \sum_{m\in M}\sum_{n\in N}\sum_{j\in J}(x_{k_{n}\left(t\right),2}\left(t\right)d_{j}^{\mathrm{par}}\\ + & x_{k_{n}\left(t\right),3}\left(t\right)\left(d_{j}^{\mathrm{lib}}+d_{j}^{\mathrm{par}}\right)+x_{k_{n}\left(t\right),5}\left(t\right)d_{j}^{\mathrm{par}}\\ + & x_{k_{n}\left(t\right),6}\left(t\right)\left(d_{j}^{\mathrm{lib}}+d_{j}^{\mathrm{par}}\right)), \end{array}$$

where the service ready to be trained is arranged to be transmitted from timeslot *t* to timeslot $t+T_{j}^{\mathrm{int}}-\left(\left(t-t_{j}\right)\mathrm{mod}T_{j}^{\mathrm{int}}\right)$.

The pseudo-code of LLUC is shown in Algorithm 2. The complexity of the adaptive learning penalty weight method, dequeued rate update mechanism, and freshness-aware transmitting method are O(|N|), O(|M||N||J|), and O(|M||N||J|), respectively.
**Algorithm 2** Lyapunov optimization-based learning and update control module (LLUC).**Require**:enqueued rate $d^{\mathrm{enq}}\left(t\right)$, initial queue backlog Q(1), transformation coefficient β, initial bandwidth penalty weight V(1), learning rate of penalty weight ζ, given threshold of penalty weight $\epsilon^{\mathrm{thr}}$, given training round proportion η**Ensure**:dequeued rate $d^{\mathrm{deq},\prime }\left(t\right)$1:**for** t=1 to *T* **do**2:   Update V(t) based on the adaptive learning penalty weight method according to (71).3:   Calculate $d^{\mathrm{deq},*}\left(t\right)$ based on the Lyapunov optimization according to (70).4:   **if** $V\left(t\right)>\epsilon^{\mathrm{thr}}$ **then**5:     Update $d^{\mathrm{deq},\prime }\left(t\right)$ and $d^{\mathrm{deq},\prime }\left(t+1\right)$ based on the dequeued rate update mechanism according to (72) and (73), respectively.6:   **end if**7:   **if** $\left(\left(t-t_{j}\right)\mathrm{mod}T_{j}^{\mathrm{int}}\right)>\eta T_{j}^{\mathrm{int}}$ **then**8:     Update $d^{\mathrm{deq},\prime }\left(t\right)$ and $d^{\mathrm{deq},\prime }\left(t+T_{j}^{\mathrm{int}}-\left(\left(t-t_{j}\right)\mathrm{mod}T_{j}^{\mathrm{int}}\right)\right)$ based on the freshnessaware transmitting method according to (74) and (75), respectively.9:   **end if**10:**end for**11:**return** $d^{\mathrm{deq},\prime }\left(t\right)$.

### 4.3. KM Algorithm-Based Channel Division Fetching Module (KCDF)

Since the application server transmits with the MEC servers or mobile devices under the cellular network, the cellular channel matching is crucial to reduce the total transmission time of requested service data $d^{\mathrm{deq},\prime }\left(t\right)$.

The total transmission time of the dequeued requested service data can be denoted as

(76) $$\begin{array}{cc} \;\;\;\;\;T^{\mathrm{fet}}\left(t\right)= & \sum_{n\in N(t)}(\sum_{m\in M(t)}(x_{k_{n}\left(t\right),2}\left(t\right)T_{0,m,j}^{\mathrm{par}}\\ & +x_{k_{n}\left(t\right),3}\left(t\right)\left(T_{0,m,j}^{\mathrm{lib}}+T_{0,m,j}^{\mathrm{par}}\right))+x_{k_{n}\left(t\right),5}\left(t\right)T_{0,n,j}^{\mathrm{par}}+x_{k_{n}\left(t\right),6}\left(t\right)\left(T_{0,n,j}^{\mathrm{lib}}+T_{0,n,j}^{\mathrm{par}}\right)), \end{array}$$

where M(t) and N(t) are the responding sets of MEC servers and mobile servers based on the dequeued service data, respectively.

First, we divide the total allocated bandwidth into two parts, one is allocated for transmitting cases 2 and 5, and another is allocated for cases 3 and 6 with more transmitted data. The allocated bandwidth divided method is designed as

(77) $$\begin{array}{cc} b_{0}^{\mathrm{par}}\left(t\right)= & b_{0}\left(t\right)\sum_{n\in N(t)}\left(\sum_{m\in M(t)}x_{k_{n}\left(t\right),2}\left(t\right)d_{j}^{\mathrm{par}}+x_{k_{n}\left(t\right),5}\left(t\right)d_{j}^{\mathrm{par}}\right)\\ & /\sum_{n\in N(t)}(\sum_{m\in M(t)}\left(x_{k_{n}\left(t\right),2}\left(t\right)d_{j}^{\mathrm{par}}+x_{k_{n}\left(t\right),3}\left(t\right)\left(d_{j}^{\mathrm{lib}}+d_{j}^{\mathrm{par}}\right)\right)\\ & +x_{k_{n}\left(t\right),5}\left(t\right)d_{j}^{\mathrm{par}}+x_{k_{n}\left(t\right),6}\left(t\right)\left(d_{j}^{\mathrm{lib}}+d_{j}^{\mathrm{par}}\right)), \end{array}$$

and

(78) $$b_{0}^{\mathrm{lib},\mathrm{par}}\left(t\right)=b_{0}\left(t\right)-b_{0}^{\mathrm{par}}\left(t\right),$$

where $b_{0}^{\mathrm{par}}\left(t\right)$ and $b_{0}^{\mathrm{lib},\mathrm{par}}\left(t\right)$ are the total allocated bandwidths of cases 2 and 5 and cases 3 and 6, based on their total transmitted data sizes, respectively.

Take cases 2 and 5 as an example, we defined the response set as $S=M^{\mathrm{par}}\left(t\right)\cup N^{\mathrm{par}}\left(t\right)$, where S is indexed by *s* and has cardinal number |S|, $M^{\mathrm{par}}\left(t\right)$ and $N^{\mathrm{par}}\left(t\right)$ are the responding sets with cases 2 and 5 of the MEC servers and mobile devices, respectively. Let A={1,⋯,|A|} be the set of |A| cellular channels indexed by *a*. The matching decision of *s* and *a* can be defined as $x_{s,a}^{\mathrm{mat}}\left(t\right)=1$ if *a* is allocated to *s*; otherwise, $x_{s,a}^{\mathrm{mat}}\left(t\right)=0$, and it is constrained by: $\sum_{s\in S}x_{s,a}^{\mathrm{mat}}\left(t\right)=1,\forall a\in A$, $\sum_{a\in A}x_{s,a}^{\mathrm{mat}}\left(t\right)=1,\forall s\in S$, where each cellular channel is allocated for, at most, one MEC server or mobile device, and each MEC server or mobile device is assigned, at most, one cellular channel. Thus, the transmission latency of the service parameter from the application server to the MEC server or mobile device *s* over cellular channel *a* is:

(79) $$T_{s,a}^{\mathrm{par}}=\frac{d_{j(s)}^{\mathrm{par}}}{\frac{b_{0}^{\mathrm{par}}\left(t\right)}{\left|S\right|}\log\left(1+\frac{p_{s,a}\left(t\right)h_{s,a}\left(t\right)}{\sigma^{2}+I_{s,a}\left(t\right)}\right)},$$

where j(s) is the service type transmitted for *s*, $p_{s,a}\left(t\right)$, $h_{s,a}\left(t\right)$, and $I_{s,a}\left(t\right)$ are the transmission power, channel gain, and co-channel interference under channel *a* to *s*, respectively.

Here, we formally formulate the problem to minimize the total transmission time of the service parameters:

(80) $$\min_{x_{s,a}^{\mathrm{mat}}\left(t\right)}Z_{3}=\frac{1}{\left|S\right|}\sum_{s\in S}\sum_{a\in A}x_{s,a}^{\mathrm{mat}}\left(t\right)T_{s,a}^{\mathrm{par}}$$

(81) $$s.t.\;\sum_{s\in S}x_{s,a}^{\mathrm{mat}}\left(t\right)=1,\forall a\in A,$$

(82) $$\sum_{a\in A}x_{s,a}^{\mathrm{mat}}\left(t\right)=1,\forall s\in S,$$

(83) $$x_{s,a}^{\mathrm{mat}}\left(t\right)\in \left\{0,1\right\},\forall s\in S,a\in A,$$

where $x_{s,a}^{\mathrm{mat}}\left(t\right)$ is the optimization variable. (81)–(83) are the constraints of the matching decision.

We leverage the KM algorithm [28] to solve the problem. The complete weighted link bipartite graph is defined as G=(S,A,<S,A>), where S and A are vertex sets of two sides, <S,A> is the link set, and the weight of its element is derived from our devised link-initialized method:

(84) $$w_{s,a}=\left\{\begin{array}{cc} T_{s,a}^{\mathrm{par}}, & \mathrm{if}\;T_{s,a}^{\mathrm{par}}<\theta \min_{a\in A}T_{s,a}^{\mathrm{par}},\\ 0, & \mathrm{otherwise}, \end{array}\right.$$

where θ is a coefficient of the minimum service data transmission time to remove the unacceptable transmission time. The feasible vertex label is satisfied: $w_{s}+w_{a}\leq w_{s,a}$, where $w_{s}=\min_{a\in A}w_{s,a}$ and $w_{a}=0$ are the vertex label of *s* and *a* in the KM algorithm. Let $G^{\mathrm{mat}}=\left(S,A,<S^{\mathrm{mat}},A^{\mathrm{mat}}>\right)$ be the equalling matching subgraph, satisfying $w_{s}+w_{a}=w_{s,a}$, where the link set $<S^{\mathrm{mat}},A^{\mathrm{mat}}>$ is initialized to an empty set.

The perfect matching of the equalling matching subgraph $G^{\mathrm{mat}}$ can be denoted as $M^{*}$, and we have the following theorem.

**Theorem 2.**  Assuming $w_{s}$ and $w_{a}$ are the feasible vertex labels, if the equalling match subgraph $G^{\mathrm{mat}}$ has a perfect matching, $M^{*}$, $M^{*}$ is also a perfect match with a minimum total weight of G.

**Proof.**  The proof is analyzed in Appendix A.    □

The steps of the KM algorithm can be elaborated as follows:

1. Initialize $w_{s}$, $w_{a}$, and $w_{s,a}$.
2. Enumerate s∈S, find a∈A satisfies $w_{s}+w_{a}=w_{s,a}$ based on the Hungarian algorithm.
3. If $a\in \left(A-A^{\mathrm{mat}}\right)\cap A^{\mathrm{rea}}$, add <s,a> into $G^{\mathrm{mat}}$; otherwise, calculate the matching distance $z=\min_{a}\left\{w_{s,a}-w_{s}-w_{a},s\in S^{\mathrm{rea}},a\in \left(A-A^{\mathrm{mat}}\right)\right\}$, set $w_{s}=w_{s}+z,s\in S^{\mathrm{rea}}$ and $w_{a}=w_{a}-z,a\in A^{\mathrm{rea}}$. Then, change the reachable path into links, e.g., $s,<a_{1}s^{*}>,a^{*}$ to $<sa_{1}>,<s^{*}a^{*}>$.
4. Repeat 2 and 3 until obtaining $M^{*}$ of $G^{\mathrm{mat}}$.

Therein, $S^{\mathrm{mat}}$ and $A^{\mathrm{mat}}$ are the vertex sets of two sides, whose elements have links in $G^{\mathrm{mat}}$, respectively. $S^{\mathrm{rea}}$ and $A^{\mathrm{rea}}$ are the searching reachable path sets of two sides by the breadth-first search in the Hungarian algorithm [29], respectively. $s^{*}$ and $a^{*}$ are the corresponding variables of *z*. The matching decision can be derived from:

(85) $$x_{s,a}^{\mathrm{mat}}\left(t\right)=\left\{\begin{array}{cc} 1, & \mathrm{if}\;<s,a>\in M^{*},\\ 0, & \mathrm{otherwise}, \end{array}\right.$$

After obtaining the result of the KM algorithm, a few individual MEC servers or mobile devices are allocated an unsatisfied cellular channel, which significantly delays the transmission time. We further design a worst-case arranging mechanism to deal with it:

(86) $$\;\;\;\;\;x_{s,a}^{\mathrm{mat}}\left(t\right)=\left\{\;\;\;\begin{array}{cc} 0,\;\;\; & \mathrm{if}\;w_{s,a}|<\;s,a\;>\in M^{*}\;=\;\max_{a\in A}w_{s,a},\;\;\\ 1,\;\;\; & \mathrm{otherwise}, \end{array}\right.$$

if $x_{s,a}^{\mathrm{mat}}\left(t\right)=0$, *s* is scheduled to be allocated, a cellular channel in the next timeslot, which consumes less time when it is allocated a satisfied channel in the next timeslot.

The pseudo-code is shown in Algorithm 3. The complexity of the naive KM algorithm is $O({\left|S\right|}^{4})$, and the KM algorithm with the slack array is $O({\left|S\right|}^{3})$. The complexities of the allocated bandwidth divided method, link-initialized method, and worst-case arranging mechanism are O(|M(t)||N(t)|), O(1), and O(1), respectively. Parts of cases 3 and 6 can be similarly solved in Algorithm 3.
**Algorithm 3** KM algorithm-based channel-division fetching module (KCDF).**Require**:total allocated bandwidth $b_{0}^{\mathrm{par}}\left(t\right)$, responding set S, cellular channel set A, link threshold ${\bar{T}}_{s,a}^{\mathrm{par}}$, transmission power $p_{s,a}\left(t\right)$, channel gain $h_{s,a}\left(t\right)$, co-channel interference $I_{s,a}\left(t\right)$**Ensure**:matching decision $x_{s,a}^{\mathrm{mat}}\left(t\right)$1:Calculate $T_{s,a}^{\mathrm{par}}$ based on the allocated bandwidth divided method according to (79).2:Initialize G based on the link-initialized method according to (84).3:Obtain the perfect matching $M^{*}$ based on the KM algorithm.4:Derive the matching decision $x_{s,a}^{\mathrm{mat}}\left(t\right)$ according to (85).5:Rearrange the matching decision $x_{s,a}^{\mathrm{mat}}\left(t\right)$ based on the worst-case arranging mechanism according to (86). 6:**return** $x_{s,a}^{\mathrm{mat}}\left(t\right)$.

## 5. Evaluation

### 5.1. System Implementation

For system implementation, we implement the framework in a real-world collaborative edge system testbed that consists of a Raspberry Pi4 Model B board (with 1.5 GHz CPU, 4 GB memory) and a desktop (with an Intel 8 Cores i7-10700F 2.90 GHz CPU and 16 GB memory). Raspberry Pi serves as the application server. The desktop serves as the MEC servers and mobile devices. All devices are connected under a local wireless router. We use the transmission control protocol (TCP) socket programming for guaranteeing reliable communication over all devices in the environment.

### 5.2. Case Study

We present a simulation of the proposed framework on the edge system testbed through a real-world image analysis case study: automatic license plate recognition. In particular, we leverage the convolutional neural network (CNN) framework as a service developed in [30]: an ImageNet model VGG-16. The VGG-16 model is a deep CNN with 16 layers for image recognition tasks and is trained in a distributed machine learning style. We use the open-source automatic license plate recognition dataset (available online: https://platerecognizer.com (accessed on 6 May 2022)) to emulate the tasks generated by mobile devices.

### 5.3. Experiment Setup

We use simulations to compare the performance of the framework. The hyperparameters of the simulation are as follows: the input size of the task is in [2,10]MB, the computation amount in [1000, 50,000] cycles, the MEC servers and mobile device number are in {10,20,30,40}, the learning rate of the penalty function is in {0.1,0.2,0.3,0.4}, the proportion of the training round is in {0.8,0.85,0.9,0.95}, and the link-initialized coefficient is in {1.5,1.8,2.1,2.4}.

Hence, we first choose some representative baselines compared with the LMKO module.

- **Fresh cache offloading priority (FCOP)**: An algorithm where the mobile device searches a MEC server with a fresh parameter cache and immediately offloads the task.
- **Cache offloading priority (COP)**: An algorithm where the mobile device searches a MEC server with cache and immediately offloads the task.
- **Offloading priority (OP)**: An algorithm where the mobile device searches a MEC server and immediately offloads the task.
- **Local execution with fresh cache priority (LEFC)**: An algorithm where the mobile device executes the task locally if it maintains a fresh parameter cache; otherwise, it offloads the task to a MEC server.

Moreover, we further pick competitive baselines compared with the LLUC module.

- **Queue backlog priority (QBP)**: An algorithm constrains the penalty weight in a relatively low range of the Lyapunov optimization.
- **Total bandwidth priority (TBP)**: An algorithm constrains the penalty weight in a relatively high range.
- **Queue backlog empty (QBE)**: An algorithm fixes the penalty weight to 0 of the Lyapunov optimization.
- **Fixed total bandwidth (FTB)**: An algorithm fixes the penalty weight in an extremely high value.

We also select a few representative strategies compared with the KCDF module.

- **Hungary algorithm (HA) [29]**: An algorithm is leveraged to solve the maximal matching problem of a non-weight bipartite graph.
- **Channel bandwidth allocated-based size (CBAS)**: An algorithm where the total bandwidth is allocated based on the responding service data size.
- **Channel bandwidth allocated-based case (CBAC)**: An algorithm where the total bandwidth is allocated based on the requesting offloading case.
- **Uniform allocation of channel bandwidth (UACB)**: An algorithm where the total bandwidth is allocated uniformly.

### 5.4. LLUC Evaluation

**Figure 2:**
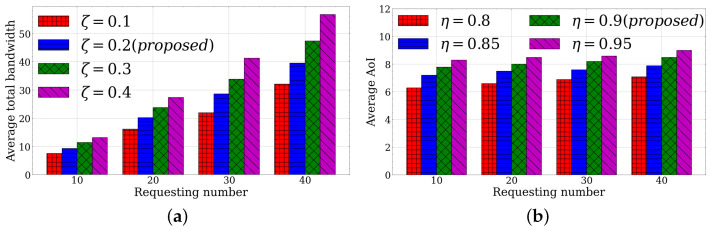
LLUC evaluation under different learning rates and training round proportions. (**a**) Learning rate. (**b**) Proportion of training round.

We first investigate the LLUC module to compare the performance of the time average–total bandwidth under different learning rates of the penalty weight. From Figure 2a, it can be shown that our proposed LLUC module with ζ=0.1 achieves the best result over the change of the requesting number. As ζ increases from 0.1 to 0.4, the performance degrades over all of the requesting numbers. A lower learning rate results in a relatively high penalty weight and the Lyapunov optimization minimizes the penalty. In the meantime, selecting a lower learning rate may lead to a higher backlog and further delay the response fetching time. Therefore, it is advisable to make a moderate selection to balance the bandwidth consumption and time overhead. Since using ζ=0.2 only increases bandwidth by 24.0% and decreases the queue backlog by 53.3% compared to using ζ=0.1 for 10 requests, we take ζ=0.2 as the learning rate, considering the bandwidth and time.

Then, we studied the LLUC module to compare the performance of the average AoI of each response service data under distinct round proportions of request rearrangements. In Figure 2b, the simulation results show that when η=0.8, i.e., when a 20% interval is left until the next training, the algorithm consistently achieves the lowest AoI regardless of the number of requests. As η increases from 0.8 to 0.95, the average AoI becomes higher. More requests are rearranged to another timeslot for service data with lower AoI. However, the time latency can deteriorate while the response timeslots are delayed. Therefore, considering the responding transmission time and AoI service data, which decrease the service fetching frequencies, we selected a medium proportion η=0.9 to balance the trade-off, whose average AoI only increases by 23.8% and the time latency decreases by 36.2%, when comparing η=0.8 under 10 requests.

### 5.5. KCDF Evaluation

**Figure 3:**
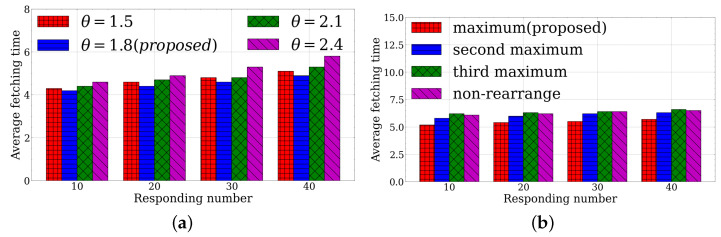
KCDF evaluation under different link-initialized coefficients and rearrange conditions. (**a**) Link-initialized coefficient. (**b**) Rearrange condition.

From the perspective of the module KCDF, we first evaluate the performance of the average fetching time under different link-initialized coefficients. Figure 3a plots that the KCDF with θ=1.8 outperforms other link-initialized coefficients as the responding number increases. It is concluded that a lower link-initialized coefficient can remove more unsatisfied links in the KM algorithms. As θ increases from 1.8 to 2.4, the average fetching time becomes higher. However, selecting a link-initialized coefficient that is too low such as θ=1.5 can increase the probability that the MEC server or mobile device fails to find a link in the equalling matching subgraph, which can significantly decline the performance. To make a feasible balance trade-off, we select θ=1.8 to guarantee the transmission time latency with at least a 2.3% performance improvement over the second-best result from θ=1.5 in 10 responses.

Secondly, we compare the average fetching time under distinct rearrange conditions in Figure 2b. It is illustrated that rearranging based on the link weight is not less than the maximum, i.e., $w_{s,a}|<\;s,a\;>\in M^{*}\;=\;\max_{a\in A}w_{s,a}$ has the minimum average fetching time when the responding number varies. The fetching time increases from the maximum, the second maximum, and the third maximum. When the application server rearranges based on the weight of the link is not less than the third, its result is even worse than the non-rearrangement. If the link is not the worst choice of the MEC server or mobile device, it is better to respond at this timeslot; otherwise, it suffers a higher fetching time. We choose the rearranging condition if the link weight is not less than the maximum to the LLUC module, which results in a time reduction of at least 3.7% compared to the second maximum case for under 10 responses.

### 5.6. Performance Comparison

#### 5.6.1. Average Cost Comparison

**Figure 4:**
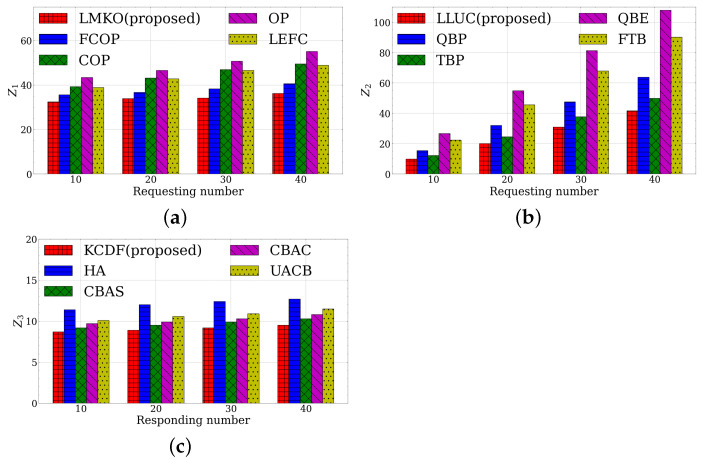
Performances under different module baselines. (**a**) LMKO baselines. (**b**) LLUC baselines. (**c**) KCDF baselines.

From Figure 4a, we investigate the average cost of the distinct baselines of the LMKO modules. Our LMKO module achieves the minimum of $Z_{1}$ under different requesting numbers. The LMKO module is capable of obtaining the minimum cost by offloading the task to the best MEC server or local execution. The second-best result belongs to the FCOP algorithm since the mobile device chooses a MEC server with a fresh cache to offload. The performances of the COP and OP are poor owing to their extra service fetching times. The worst result is brought by the LEFC since it does not take advantage of offloading in the edge system. The LMKO module is efficient in terms of the cost of the time delay and energy consumption with at least a 4.1% performance gain compared to the second-best result from FCOP (for under 10 requests).

#### 5.6.2. Average Total Bandwidth Comparison

Figure 4b illustrates the performance of the time-averaged total allocated bandwidth of the baselines of the LLUC baselines. The proposed LLUC module has a superior result comparing other baselines while the requesting number increases. The efficiency of the LLUC module maintains a controlled queue backlog while minimizing the total allocated bandwidth. In the meanwhile, the TBP algorithm obtains the second minimum result since it takes a higher penalty weight but causes a longer queue backlog. The QBP algorithm preferentially considers the queue backlog, leading to a medium result. The FTB algorithm delivers a high performance despite a fixed total bandwidth, due to its large backlog. The worst result belongs to the QBE algorithm, which keeps the 0 queue backlog, even as the bandwidth significantly increases. The LLUC module outperforms other baselines in regard to queue backlog stability and total allocation bandwidth with at least a 19.7% performance gain compared to the second-best result from TBP under 10 requests.

#### 5.6.3. Average Fetching Time Comparison

Figure 4c displays the results of comparing the average fetching times of the baseline methods of the KCDF module. Our KCDF module exhibits superior performance across varying response numbers. The KCDF module finds a perfect match in the equalling matching subgraph, where each MEC server or mobile device matches its best-allocated cellular channel. The second lowest number belongs to the CBAS algorithm, which allocates the bandwidth according to the service data size. Each MEC server or mobile device can obtain the satisfied channel. The CBAC algorithm allocating the bandwidth with the offloading case suffers a similar situation with the CBAS and attains a medium result. The UACB has a poor result since it uniformly allocates the bandwidth leading to the response with service libraries and parameters having unsatisfied transmission latency. The HA algorithm suffers the worst result since it never updates the vertex label while it cannot find a link in the equalling matching subgraph, which results in a few vertices being matched, i.e., a few channels are allocated. Thus, our KCDF module has efficient performance in terms of average fetching time with at least a 2.2% performance gain compared to the second-best result from CBAS under 10 responses.

#### 5.6.4. Average Time Cost of Baselines Combination

**Figure 5:**
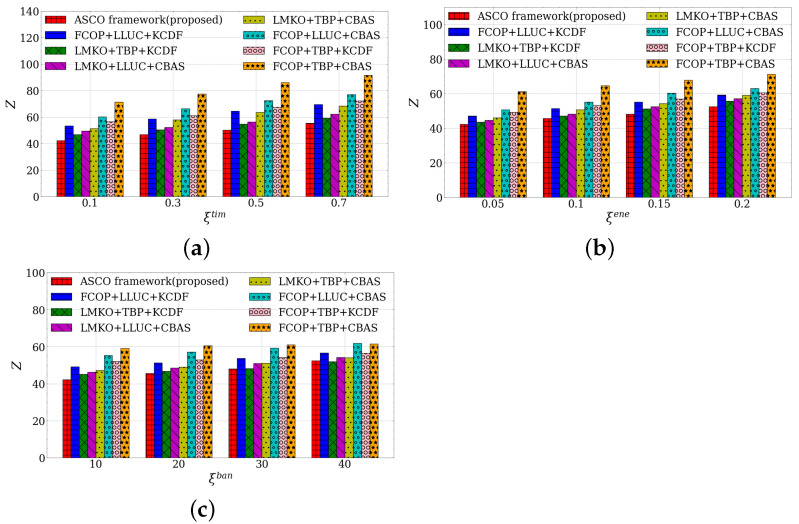
Performances under different baseline combinations. (**a**) Average time cost. (**b**) Average energy cost. (**c**) Average bandwidth consumption.

In Figure 5a, we investigated the performance of the average time cost of the baseline combinations, which includes our proposed ASCO framework (LMKO, LLUC, and KCDF modules) and other baselines achieving the competitive result consisting of FCOP, TBP, and CBAS algorithms. We can see that our ASCO framework always outperforms other baseline combinations while the time weight parameter changes, and the weights of energy and bandwidth remain. We minimize the average time cost by finding the most suitable offloading decision and allocating the best cellular channel. Other baseline combinations have declined results comparing our framework. LMKO+TBP+KCDF, LMKO+LLUC+CBAS, and LMKO+TBP+CBAS achieved moderate performance, as there was not a significant improvement in the modules. On the other hand, FCOP+LLUC+KCDF, FCOP+LLUC+CBAS, and FCOP+TBP+KCDF incurred higher costs, as their modules placed less emphasis on time concerns. Take FCOP+TBP+CBAS as an example, it had the worst performance due to the absence of the proposed modules. As $\xi^{\mathrm{tim}}$ increased, the performance gap between our framework and another baseline combination enlarged, which explains why the proposed framework has a significant gain in terms of time delay with at least a 9.6% improvement compared to the second-best result from LMKO+TBP+KCDF under $\xi^{\mathrm{tim}}=0.1$.

#### 5.6.5. Average Energy Cost of Baselines Combination

Figure 5b shows the performance of the average energy cost of the combinations. Our ASCO framework keeps the best result while the energy weight varies and the weights of the time and bandwidth are fixed. The LMKO module makes an economized energy offloading decision to save the energy consumption of mobile devices. At the same time, other baseline combinations with the LMKO module outperform other combinations without the LMKO module due to the consideration of energy in the LMKO module. LMKO+TBP+KCDF, LMKO+LLUC+CBAS, and LMKO+TBP+CBAS obtain middle performances due to the lack of energy concerns. FCOP+LLUC+KCDF, FCOP+LLUC+CBAS, and FCOP+TBP+KCDF have higher costs since their modules are not efficient in terms of costs. Similarly, FCOP+TBP+CBAS had the worst performance. Hence, the average energy result verifies that our proposed framework achieves superior performance with respect to energy consumption with at least a 2.8% improvement compared to the second-best result from LMKO+TBP+KCDF under $\xi^{\mathrm{ene}}=0.05$.

#### 5.6.6. Average Bandwidth Consumption of Baselines Combination

Figure 5c illustrates the comparison of the average bandwidth consumption allocated from the application server under the baseline combinations. The proposed ASCO framework attains minimum results except in an extreme case with $\xi^{\mathrm{ban}}=40$, while the weights of the time and energy are fixed. In this case, the emphasis on bandwidth allocation is extremely significant so that our framework only obtains the second-best performance while LMKO+TBP+KCDF has the best result. LMKO+LLUC+CBAS and LMKO+TBP+CBAS obtain middle performances because they do not well balance the total bandwidth and mobile device cost. FCOP+LLUC+KCDF, FCOP+LLUC+CBAS, FCOP+TBP+KCDF, and FCOP+TBP+CBAS always obtain the worse results due to the algorithm’s inefficiency. However, the value of the bandwidth is impractical since it leads to relatively less consideration of the time delay and energy consumption. In most moderate-weight cases, our framework dominates other baseline combinations in regard to the average bandwidth consumption with at least a 6.2% improvement compared to the second-best result from LMKO+TBP+KCDF under $\xi^{\mathrm{ban}}=10$.

## 6. Conclusions

In our work, we consider a scenario of AoI-aware service caching-assisted offloading. The proposed ASCO framework consists of three modules: (1) the LMKO module based on the method of Lagrange multipliers with KKT conditions. (2) The LLUC module based on the Lyapunov optimization. (3) The KCDF module based on the KM algorithm. The simulation results verify that the proposed ASCO framework outperforms other baseline combinations with respect to time overhead, energy consumption, and allocated bandwidth. The ASCO framework is efficient in the individual inference task and global bandwidth allocation and is viable to be practically deployed.

This work can be extended in several future directions. First, considering the proactive service caching, the MEC server can predict the offloading request and call for the application server for advanced fetching. Second, considering the task partition, if the tasks are partitioned before execution, the subtasks can be executed in distinct MEC servers or locally.

## Data Availability

Not applicable.

## Appendix A

Here is the proof of Theorem 2 from Section 4.3.

**Proof.** On the one hand, as $G^{\mathrm{mat}}$ is a generated equalling matching subgraph of G, the perfect matching $M^{*}$ of $G^{\mathrm{mat}}$ is also a perfect matching of G, and $\sum_{<s,a>\in M^{*}}w_{s,a}=\sum_{s\in S}w_{s}+\sum_{a\in A}w_{a}$. On the other hand, any non-perfect matching *M* has $\sum_{<s,a>\in M}w_{s,a}\leq \sum_{s\in S}w_{s}+\sum_{a\in A}w_{a}$. Therefore, the perfect matching $M^{*}$ has a minimum total weight equaling its upper bound. □
